# Computational Approaches for Designing Heterostructured Electrocatalysts

**DOI:** 10.1002/smsc.202400544

**Published:** 2025-02-11

**Authors:** Miyeon Kim, Kyu In Shim, Jeong Woo Han

**Affiliations:** ^1^ Department of Materials Science and Engineering Research Institute of Advanced Materials Seoul National University Seoul 08826 Republic of Korea

**Keywords:** activities, computational materials sciences, density functional theories, electrocatalysts, heterostructures, modeling, stabilities

## Abstract

Electrocatalysts for oxidation and reduction reactions are crucial for sustainable energy production and carbon reduction. While precious metal catalysts exhibit superior activity, reducing reliance on them is necessary for large‐scale applications. To address this, transition metal‐based catalysts are studied with strategies to enhance catalytic performance. One promising strategy is heterostructures, which integrate multiple materials to harness synergistic effects. Developing efficient heterostructured electrocatalysts requires understanding their intricate characteristics, which poses challenges. While *in* *situ* and *operando* spectroscopy provides insights, computational materials science is essential for capturing reaction mechanisms, analyzing the origins at the atomic scale, and efficiently exploring innovative heterostructures. Despite growing recognition of computational materials science, standardized criteria for these systems remain lacking. This review consolidates case studies to propose approaches for modeling and analyzing heterostructures. It categorizes heterostructure types into vertical, semivertical, and lateral, defines their characteristics, and propose insights into minimizing or exploiting strain effects from lattice mismatches. Furthermore, it summarizes computational analyses of heterostructure stability and activity across reactions, including oxygen evolution, hydrogen evolution, oxygen reduction, carbon dioxide reduction, nitrogen reduction, and urea oxidation. This review provides an overview to refine heterostructure designs and establish a framework for systematic modeling and analysis to develop efficient electrocatalysts.

## Graphical abstract

This review systematically explores computational approaches for modeling and analyzing heterostructures. From a computational materials science perspective, it provides insights into determining heterostructure design types, optimizing lattice strain, and analyzing structural and electronic properties through case studies. Applications of heterostructured electrocatalysts and machine learning methods are discussed, contributing to the designing of heterostructure materials for sustainable energy systems.

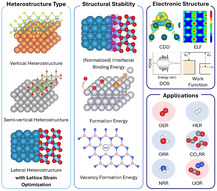

## 1 Introduction

Electrocatalysis is essential for advancing sustainable energy production and efficient carbon reduction technology.^[^ ^1^ ^]^ Efforts to enhance catalytic efficiency are imperative across various electrochemical oxidation and reduction reactions. Notably, electrocatalysts play a pivotal role in water splitting, an ecofriendly method for hydrogen production without carbon emissions, which involves the oxygen evolution reaction (OER) at the anode and the hydrogen evolution reaction (HER) at the cathode. Meanwhile, enhancing the catalytic efficiency of oxygen reduction reaction (ORR) at the cathode is significant for improving the performance of fuel cells and metal–air batteries. In carbon dioxide reduction reaction (CO_2_RR), advanced electrocatalysts are instrumental in reducing greenhouse gases while yielding high‐value products. The effective nitrogen reduction reaction (NRR) and urea oxidation reaction (UOR) offer promising avenues for next‐generation sustainable energy, with potential benefits including increased ammonia production and improved fertilizer availability.^[^ ^2^ ^]^ For the industrial commercialization of these electrochemical reactions, a wide range of electrocatalysts have been explored. Platinum group metal catalysts are known to exhibit excellent performance in various electrochemical reactions. However, their high cost and limited availability necessitate alternatives for large‐scale applications. Consequently, alternative electrocatalysts, including transition metal (TM) oxides, nitrides, carbides, sulfides, and phosphides, as well as carbon‐based materials such as graphene, graphite with heteroatom doping, and hexagonal boron nitride (hBN), have been used in electrochemical reactions.^[^ ^3^ ^]^ Despite the potential of these alternatives, significant opportunities for the improvement in catalytic activity and durability still exist. In addition to exploring new material groups, several strategies aimed at enhancing the intrinsic properties of catalysts have been proposed. These include nanostructure engineering, vacancy engineering, phase engineering, interface engineering, structure integration, doping, and strain effects.^[^ ^4^ ^]^ The adoption of these approaches has demonstrated that cost‐effective TM‐based electrocatalysts can achieve or even surpass the performance of precious metal catalysts. This offers a promising strategy for reducing costs without compromising efficiency.

Among these various strategies, heterostructure catalysts, also referred to as heterogeneous structures, which combine multiple materials to significantly enhance performance, have garnered particular attention. Catalysts composed of a single material often face a trade‐off between activity and durability.^[^ ^5^ ^]^ To address this, the heterostructure approach has been applied, wherein multiple materials are integrated to complement each other's limitations.^[^ ^6^ ^]^ For example, when a metal and a semiconductor form a heterojunction, a built‐in electric field is established due to differences in work function and electronic band structure between the two materials. This electric field can induce a Mott–Schottky heterojunction, enhancing electron transfer efficiency and catalytic activity.^[^ ^7^ ^]^ Additionally, phase engineering, which employs both long‐range disordered amorphous and conductive crystalline phases, has been reported to improve catalyst activity and stability.^[^ ^8^ ^]^ Low‐dimensional materials based heterostructures are also of great interest due to their tunable electronic properties, though challenges remain in developing controllable synthesis methods. Theoretical calculations and *in* *situ* characterizations have been utilized to better understand these heterostructure catalysts.^[^ ^9^ ^]^ Furthermore, surface reconstruction during electrochemical reactions can transform precatalysts into layered double hydroxides (LDH) or oxyhydroxides, forming protective layers that prevent dissolution and provide active sites. These heterostructures function as multifunctional catalysts, improving electrochemical oxidation and reduction reactions by leveraging the advantages of diverse materials.^[^ ^10^ ^]^ Finally, integrating catalysts with distinct roles is a viable strategy. For instance, in alkaline HER, both water splitting capability and efficient hydrogen adsorption are necessary, as protons are sourced from water. To meet these requirements, an integration of metal oxides, which facilitate water splitting, and zero‐valent metals, which provide active sites for hydrogen adsorption, can be employed.^[^ ^11^ ^]^ These approaches not only enable bifunctional capabilities, allowing a single catalyst to be effective across a range of reactions or at both the anode and cathode, but also harness the synergistic effects at the material interfaces, thereby overcoming the limitations of individual components. Additionally, fast electron transfer kinetics associated with heterostructure catalysts can enhance their durability. This improvement derived from the heterostructure catalysts can lead to more efficient and long‐lasting performance during electrochemical reactions.^[^ ^12^ ^]^

**Figure 1:**
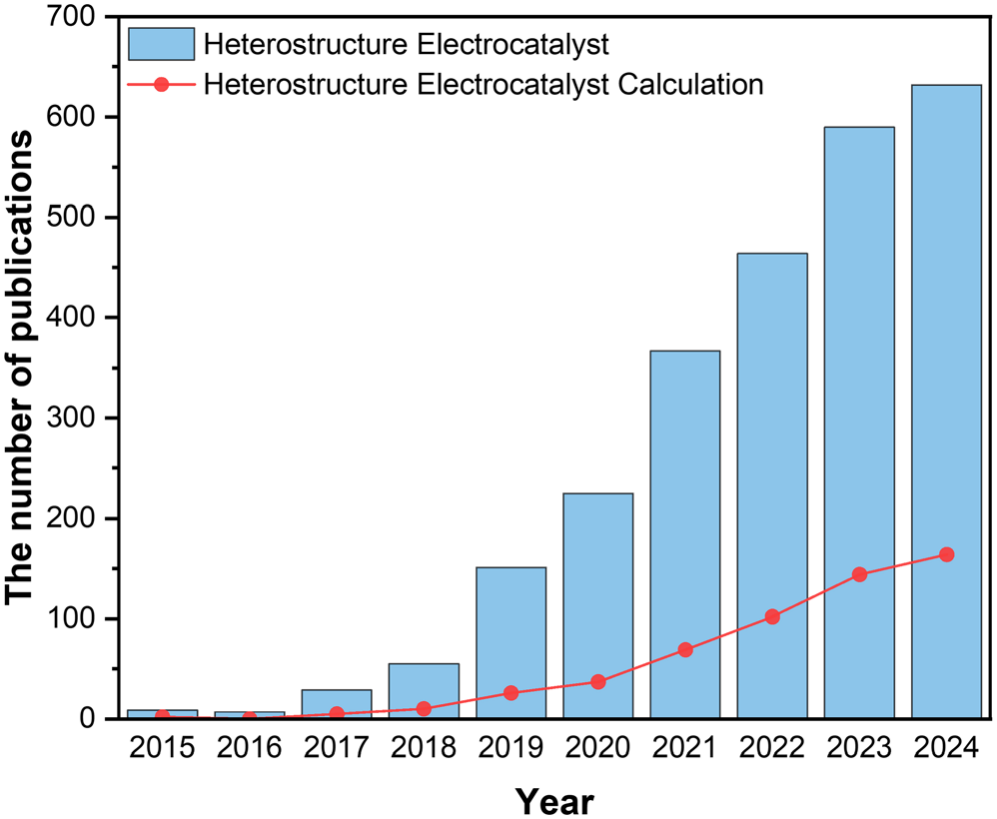
The number of publications on the topics of “Heterostructure Electrocatalyst” and “Heterostructure Electrocatalyst Calculation” from 2015 to 2024. The data for this graph were sourced from the Web of Science on January 1, 2025.

To further enhance the potential of heterostructured catalysts, it is imperative to thoroughly understand their characteristics and identify the key factors influencing their catalytic performance. Characterization techniques such as transition electron microscopy (TEM), atomic force microscopy (AFM), X‐ray diffraction, UV–vis spectroscopy, and X‐ray photoelectron spectroscopy (XPS) provide critical insights into the structural, optical, and electronic features of these catalysts. These techniques facilitate assertions regarding their composition, surface facets, oxidation states, and morphological characteristics. Furthermore, heterostructures exhibit complex systems where multiple materials interact simultaneously, making *in* *situ* and *operando* spectroscopy essential for observing real‐time characteristics.^[^ ^13^ ^]^ For example, *in* *situ* microscopy, including AFM, TEM, and scanning electron microscopy (SEM), can characterize mechanical properties and deformation process, even for materials with small size and thickness.^[^ ^14^ ^]^ *In* *situ* Raman spectroscopy enables monitoring of reaction intermediates and structural changes in real time.^[^ ^15^ ^]^ Additionally, *operando* X‐ray absorption spectroscopy elucidates oxidation states and local bonding configuration on an elemental basis.^[^ ^13^ ^]^ Despite advancements in these characterization methods, directly capturing reaction mechanisms at the atomic level remains a challenge. Specifically, it is difficult to observe electron transfer and charge distribution at interfaces, particularly during rapid electrochemical reactions or complex multistep processes. Relying solely on experimental observations can be limiting, particularly in the study of complex heterostructured electrocatalysts with numerous variables. Therefore, a collaborative approach that integrates experimental methods with computational materials science is necessary. Computational materials science has emerged as a crucial methodology, offering insights that experiments alone cannot achieve. It complements experimental challenges and suggests novel catalysts that have yet to be revealed. In line with this, research combining experimental methods with computational analysis on heterostructured catalysts has become increasingly prevalent.^[^ ^16^ ^]^ Advances in computing power have made it feasible to implement methodologies such as density functional theory (DFT), molecular dynamics (MD), and machine learning (ML). Especially, computational materials science plays a vital role by enabling the identification of active sites and mechanisms at the atomic level, thus determining the key factors that drive the progress of electrocatalysts. DFT allows for the analysis of charge transfer and electron localization function (ELF) at heterointerfaces, providing insights into electron distribution tendencies. It can also analyze work function differences that affect the directions of electron transfer and the formation of Schottky barrier. MD can examine time‐dependent interactions between molecules and the catalyst on a larger scale, offering information such as diffusion coefficient and ionic conductivity. ML is particularly advantageous for a broad exploration of diverse material groups by considering various factors. For instance, in 2D material heterostructures, it can consider variables such as rotation angles between stacking layers, interlayer spacing, and bandgap differences.^[^ ^16^ ^]^ These methods facilitate a comprehensive analysis and suggest optimal combinations that would be challenging to achieve through experiments. Therefore, the collaboration between computational and experimental approaches has become indispensable in the research of heterostructured electrocatalysts. Computational materials scientists can reveal the underlying properties of experimentally synthesized heterostructures and propose new designs that can be validated by experimentalists. Such collaborations will expedite the development of high‐performance heterostructured electrocatalysts. The increasing number of publications on “Heterostructure Electrocatalyst” and “Heterostructure Electrocatalyst Calculation” during the last 10 years (from 2015 to 2024) demonstrates the noticeable interest and research activity in these topics (**Figure** **1**).

Despite the myriad benefits of computational methods in exploring complex material systems and optimizing the catalytic performance, these methods face certain limitations. Constructing the precise heterostructure is significant in the computational approaches, yet it often relies on the researcher's individual judgment and becomes challenging due to the consideration of various variables. The absence of standardized protocols for computational analysis further complicates the process. As a result, the unique challenges in constructing heterostructured electrocatalysts necessitate systematic approaches. For example, discerning the appropriate heterostructure type depends on accurately pinpointing the active sites within the surface model of heterostructures. Implementing appropriate lattice strain is important, as the combination of materials with different lattice constants can affect the catalysts’ characteristics.^[^ ^17^ ^]^ It is also crucial to implement moderate mismatch levels that minimize lattice strain to ensure synthesizability and prevent the distortion of material properties. Furthermore, it is pivotal to carefully evaluate the stability of heterostructures and consider the phase changes and defect formation during electrochemical reactions, as these factors significantly impact the catalytic performance of heterostructures.^[9c]^ Given these complexities, a systematic approach is necessary to effectively model heterostructured electrocatalysts. Although there have been review articles on specific material groups, electrochemical reactions, experimental methods, and general computational methods for electrocatalysts, comprehensive reviews on practical computational methodologies for heterostructured electrocatalysts are not sufficient. This review aims to categorize the heterostructure types and present the systematic evaluation approaches of stability and activity, thus facilitating the integration of computational methodologies into heterostructure systems. Furthermore, we will introduce the extensive applications of heterostructures across various electrochemical reactions, including OER, HER, ORR, CO_2_RR, NRR, and UOR by focusing on the activity descriptors. Through an examination of current computational research, this review endeavors to advance the modeling and analysis of heterostructured electrocatalysts.

## 2 Construction of Heterostructure for Computational Modeling

### 2.1 Heterostructure Modeling Types

Heterostructures are formed by combining two materials with different compositions and lattice constants. These structures can be categorized either by their stacking direction into vertical and lateral heterostructures or by their bonding type into van der Waals (vdW) and covalent heterostructures.^[^ ^18^ ^]^ Additionally, heterostructures can be classified based on the materials’ dimensionality—ranging from 0D, 1D, 2D, to 3D.[^9^, ^19^] Moreover, they can be further differentiated by their phase and electrical properties, such as amorphous–crystalline and metal–semiconductor heterostructures.^[^ ^8^, ^20^ ^]^ Therefore, the classification of heterostructures can be diverse, considering factors like stacking methods, bonding types, material dimensions, and materials’ properties. On the other hand, in computational modeling, it is required to consider not only these categories but also the distinct factor inherent to computational materials science. Periodic boundary condition enables the efficient simulation of catalyst within a unit cell by repeating models to represent a large and infinite system. However, due to the intrinsic nature of this system, computational models do not directly correspond to experimental morphologies or structures in a single and definite way. Heterostructures can be constructed in a variety of ways with the periodic system. This flexibility allows researchers to select the most suitable model structure based on the specific characteristics, especially the target active sites. Therefore, computational materials scientists should carefully consider the construction factors from multiple perspectives. For a systematic approach in computational materials science, we refined the existing heterostructure types by considering the stacking direction and the degree of interaction at the interfaces, whether fully or partially interacting (Graphical Abstract). We specifically focused on the periodic unit cell system, where one material's structure is optimized to match the lattice constant of another. In addition, computational modeling requires a vacuum to analyze adsorption energy and activation barriers on the surface, as it prevents interactions between unit cells along a specific axis, which is essential for understanding catalytic activity. Without a vacuum, distinguishing vertical and lateral heterostructures becomes more complicated. Therefore, to focus on surface reaction mechanisms, we restricted our categorization to systems with a vacuum. With these frameworks, we introduced three computational heterostructure model types: vertical, semivertical, and lateral. These classifications are formulated to provide a comprehensive basis for modeling heterostructures. The characteristics of these three heterostructure types have been summarized to provide insights into selecting the most appropriate model.

#### 2.1.1 Vertical Heterostructure

Constructing vertical heterostructure involves stacking materials vertically, enabling the entire surface of materials to interact with each other. In this configuration, each material is periodically repeated along two axes, forming distinct layers. A defining feature of vertical heterostructure is that one material completely covers the surface of another. This structure type is widely used due to its ability to facilitate full interlayer interactions between the materials.

**Figure 2:**
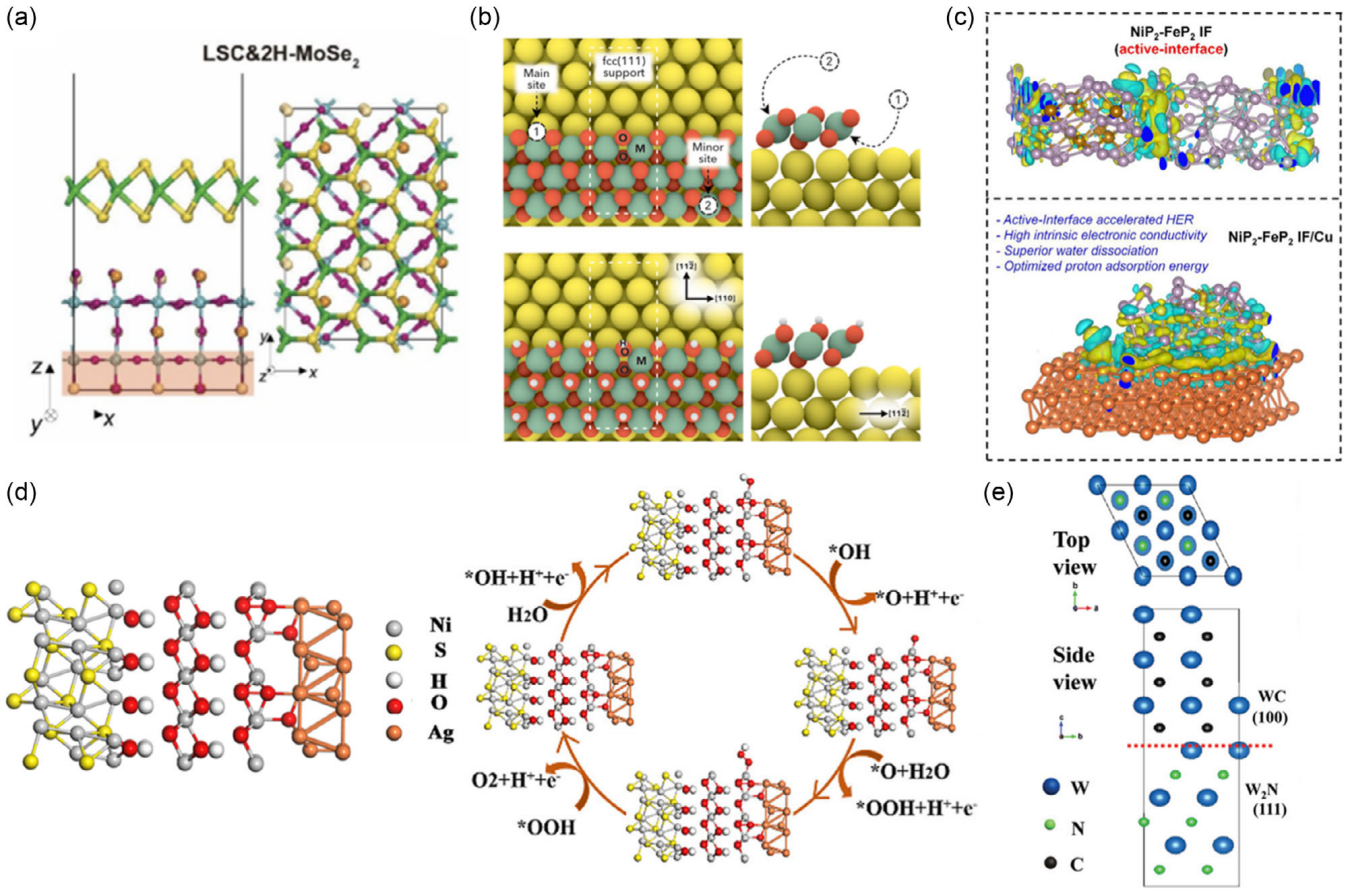
a) Vertical heterostructure modeling of 2H‐MoSe_2_/La_0.5_Sr_0.5_CoO_3−*δ*_ (LSC). Reproduced under the terms of the Creative Commons CC BY license.^[^ ^23^ ^]^ b) Semivertical heterostructure modeling of nanoribbon of MOOH on noble metal (M = Ni, Fe, Co, Mn; noble metal = Pt, Ag, Au). Reproduced with permission.^[^ ^34^ ^]^ Copyright 2019, American Chemical Society. c) Combination of lateral heterostructure and semivertical modeling of NiP_2_‐FeP_2_/Cu. Reproduced with permission.^[^ ^42^ ^]^ Copyright 2021, American Chemical Society. d) Lateral heterostructure modeling of NiOOH@Ag/Ni_3_S2. Reproduced with permission.^[^ ^45^ ^]^ Copyright 2022, American Chemical Society. e) Lattice mismatch minimization for epitaxial growth of WC/W_2_N. Reproduced with permission.^[^ ^55^ ^]^ Copyright 2019, WILEY‐VCH GmbH.

In the vertical heterostructure modeling, the active sites are generally located in the overlying materials. This modeling approach is effective for analyzing catalysts with well‐defined active sites. This concept can be utilized to investigate the synergistic effects between materials at the exposed surface. To begin with, this structure can be used to study how conductive materials enhance the electrical conductivity of 2D materials. Back et al. harnessed the high stability of hexagonal boron nitride (hBN) as an ORR catalyst and sought to improve its conductivity by forming heterostructures with metals. They constructed vertical heterostructures using DFT, focusing on hBN to thoroughly elucidate how its electronic structure and catalytic activity were altered by the influence of the metal supports and the vacancy formation.^[^ ^21^ ^]^ This strategy has been widely reported for studying the influence of metal supports on hBN in ORR and HER, utilizing vertical heterostructures.^[^ ^22^ ^]^ This approach demonstrates the effectiveness of vertical heterostructure in providing a detailed analysis of active sites within the overlying layer. Another example involves the vertical heterostructures to analyze the increase in conductivity of TM dichalcogenides (TMDs), which are widely used in electrocatalysts due to their electronic tunability. When MoSe_2_ was supported on La_1–*x*_Sr_*x*_CoO_3–*δ*_ (LSC), the vertical heterostructure modeling effectively exhibited that the phase transition from the 2H phase to the more conductive 1T phase was facilitated, allowing for better OER and HER activity (**Figure** **2**a).^[^ ^23^ ^]^ This demonstrates that vertical heterostructure efficiently revealed electronic properties and analyzed the ease of phase transitions at the overlying active sites.

Beyond these examples, vertical heterostructures have been widely applied to core–shell, encapsulated, and multimaterial catalysts, effectively elucidating how changes in active sites influence catalytic performance. Seenivasan et al. proposed a catalyst composed of a NiCo_2_S_4_ core and NiS nanoshell. The interaction between NiCo_2_S_4_ and NiS not only increased the number of active sites but also constructed a rough surface. This study effectively analyzed the OER and HER mechanisms by considering NiS as the active site. By modeling the deformation and interaction of these catalysts, this study provided notable insights into the role of NiS as the active site.^[^ ^24^ ^]^ In addition, a catalyst, synthesized through a two‐step hydrothermal reaction, formed a CoOOH nanosheet on Ni_2_P. The vertical stacking of two materials, with CoOOH considered as the active site, facilitated the analysis of OER mechanism.^[^ ^25^ ^]^ Furthermore, a strategy involving a TM phosphide encapsulated by *N*‐doped carbon was utilized to enhance alkaline HER activity. Vertical heterostructures were employed to explain the improved activity of MoP. Calculations confirmed that especially pyridinic *N*, compared to pyrrolic and graphitic *N*, played a critical role in water dissociation and hydrogen adsorption.^[^ ^26^ ^]^ Finally, the synergistic effects of cobalt's different oxidation states were demonstrated when cobalt oxide was present in two phases supported by carbon. By comparing Co_3_O_4_/CoO/C with Co_3_O_4_/C and calculating ORR mechanism specifically at the active site of Co_3_O_4_, the researchers effectively leveraged vertical heterostructure in a multilayer configuration.^[^ ^27^ ^]^ These findings highlight the versatility of vertical heterostructure construction in capturing full material interactions and uncovering the distinct characteristics of overlying active sites across diverse electrocatalyst morphologies.

Furthermore, vertical heterostructure is valuable in studying surface‐reconstructed electrocatalysts, particularly those with activated LDH or oxyhydroxides as active sites. When precatalysts are exposed to high‐pH alkaline electrolytes, they undergo surface reconstruction into (oxy)hydroxides.^[^ ^28^ ^]^ For example, FeNi alloy and FeNi oxyhydroxide vertical heterostructure were constructed to investigate the surface reconstruction of Fe_0.4_Ni_0.6_ alloys, comparing the OER activity of Ni/NiOOH and explaining the role of Fe incorporation.^[^ ^29^ ^]^ Another study utilized vertical heterostructures to examine the OER activity of surface reconstructed catalysts in Ni_3_N/Ni core with an ultrathin Ni_3_N shell. This study focused on NiOOH as the active sites in the vertical heterostructures of Ni@NiOOH and Ni_3_N@NiOOH.^[^ ^30^ ^]^ Similarly, research was conducted on the core–shell‐like structure formed by the surface reconstruction of NiCoMoN during electrochemical reaction. By comparing the OER activity of isolated NiCoOOH with that of the NiCoOOH/NiCoMoN vertical heterostructure, the superior OER activity of the heterostructure was demonstrated through DFT calculations. In this study, the vertical heterostructure effectively explained how the catalytic activity changes depending on the presence of the nitride, with NiCoOOH serving as the active site.^[^ ^31^ ^]^ This approach enables detailed comparisons of catalytic activity and reaction mechanisms in the (oxy)hydroxide phases. Vertical heterostructure effectively investigates the synergistic effects between supporting precatalysts and activated (oxy)hydroxides, facilitating in‐depth analysis of the covering material.

In vertical heterostructures, it is also possible for both the overlayer and the underlying material to serve as active sites in the opposite direction for catalytic reactions, especially when dealing with 2D materials or thin films. This is feasible because, in computational simulations, the bottom layers of the underlying material, especially for thick layers like 3D materials, are typically fixed to represent bulk properties, but the thin or 2D layers are generally fully relaxed to accurately capture surface or interface behaviors. As a result, vertical heterostructures involving only such 2D materials allow both components to act as active sites in the opposite direction. For instance, a 2D–2D heterointerface using Pt nanodendrite and NiFe LDH was examined for a HER catalyst. Pt contributed to hydrogen adsorption, while NiFe LDH facilitated water dissociation. The vertical heterostructure of 2D Pt(110) and NiFe LDH(001) revealed the electron depletion in NiFe LDH compared to its isolation model, making it more favorable for water and OH adsorption. At the same time, Pt in the heterostructure exhibited stronger dissociated water adsorption energy, indicating that the formation of the heterostructure with NiFe LDH enhanced its water dissociation ability. This example highlights the use of vertical heterostructure design to explore adsorptions at the outer surfaces of both materials, confirming that both can function as active sites.^[^ ^32^ ^]^ Additionally, a self‐assembled double‐heterojunction electrocatalyst of NiS_2_/Ni_3_C@C was analyzed by separating it into two vertical heterostructures: Ni_3_C/C and NiS_2_/Ni_3_C. The Ni_3_C/C interface exhibited efficient charge and mass transfer abilities through electronic structure analysis. To further analyze HER activity, NiS_2_/Ni_3_C was compared to individual NiS_2_ and Ni_3_C, with the adsorption site located at the interspace between two materials. This demonstrates that vertical heterostructure can effectively differentiate and analyze interfacial regions that significantly influence catalytic activity, while also considering adsorption sites at the interspace.^[^ ^33^ ^]^ Hence, these instances illustrate that vertical heterostructures can also efficiently utilize both overlayer and underlying materials as active sites, depending on the specific materials and research objectives.

#### 2.1.2 Semivertical Heterostructure

Semivertical heterostructure refers to a structure where two materials are vertically stacked, but one material partially covers the other. This strategic overlap ensures that the surfaces of both materials remain exposed in the same direction. Unlike the vertical heterostructure, in a semivertical heterostructure, the covering material is periodically repeated along only one axis, allowing both overlayer and underlying materials to remain accessible at the surface. This type facilitates the investigation of catalytic properties of each material while enabling in‐depth examinations of the direct interactions at their interface.

Semivertical heterostructure is useful for analyzing the edge sites of the covering material, which can serve as potent active sites. For example, Back et al. modeled nanoribbons of TM‐based oxides and oxyhydroxides on noble metals to improve the stability and ORR activity during electrochemical reactions (Figure 2b). TM oxides and oxyhydroxides of Co, Fe, Mn, and Ni were established with a single layer, while Ag, Au, and Pt served as support metals. Through semivertical heterostructure, the edge sites of these oxyhydroxides and oxides were exposed, allowing adsorbates to interact with both the supporting metals and the covering oxides. They distinguished between the main sites at the interface of the support and nanoribbons and the minor sites at the edge sites of the nanoribbons.^[^ ^34^ ^]^ Likewise, Wen et al. employed this heterostructure type for the Schottky heterojunction formed by nanosheet NiS and NiFe hydroxide. They constructed the heterostructure with NiFe LDH covering O‐doped NiS and conducted OER mechanism calculations at the edge sites of the LDH. This approach efficiently helped to study the edge sites of hydroxide in semivertical heterostructure electrocatalysts.^[^ ^35^ ^]^ Therefore, under periodic boundary conditions, semivertical heterostructure can be employed to model catalysts where the edge sites of the covering material are exposed by optimizing its periodicity along one axis.

Another important approach with semivertical heterostructure is the investigation of interfaces where both materials form direct bonds. A noticeable feature of semivertical heterostructure is their ability to calculate reaction mechanisms at these interfaces with direct bonds, providing insights into how both materials contribute to catalytic activity. For example, Kavinkumar et al. proposed a W_2_N_3_/Fe_2_N system for water electrolysis, using semivertical heterostructure to form a conductive interface between coral‐like 3D Fe_2_N and 2D W_2_N_3_ nanosheets. They calculated the water splitting kinetics, highlighting enhanced HER activity of the semivertical heterostructure compared to individual Fe_2_N.^[^ ^36^ ^]^ Furthermore, a combination of semivertical heterostructure and a unique strategy was applied to simulate and analyze strong electronic coupling in an alkaline HER catalyst. By partially placing a NiHO layer on Ni_2_P(111), You et al. investigated the interaction at various adsorption sites, including the edge sites of NiHO, the NiHO/Ni_2_P interface, and the surface of Ni_2_P. Additionally, to investigate the effect of a more negative charge on the catalytic performance of Ni_2_P, they substituted part of the Ni_2_P layer with NiHO. This unique approach led to increased electron accumulation on Ni_2_P, and DFT calculations revealed that the negatively charged phosphide significantly enhanced HER activity. They illustrated their novel approach to introducing negative charge.^[^ ^37^ ^]^ In addition to this example, Zhai et al. used semivertical heterostructure and atom replacement to explore the synergy between bimetallic oxide and sulfide. They established a Ni_3_S_2_/MoS_2_ semivertical heterostructure where a 2D nanosheet formed an interface with a 1D nanorod. To simulate oxides, they substituted sulfur atom at the interface of MoS_2_ edge and Ni_3_S_2_ surface with oxygen atom and created a NiMoO_x_/NiMoS catalyst. This structure showed the thermoneutral hydrogen adsorption energy and a lower OER overpotential compared to the Ni_3_S_2_/MoS_2_ system. They suggested that the combination of Ni and Mo in this heterostructure enhanced bifunctional catalytic activity.^[^ ^38^ ^]^ Similarly, Chen et al. applied semivertical heterostructure to model a heterostructure composed of tungsten (W) nanoparticles and WO_2_ nanorods, another example of a system incorporating 1D material. High‐magnification SEM and energy‐dispersive X‐ray spectroscopy confirmed the 1D nanorod structure. In this system, WO_2_ acted as a Lewis acid, facilitating water adsorption, dissociation, and proton storage, while W served as a Brønsted acid site for proton desorption. Constructing the semivertical heterostructure suggested the synergistic effects of W and WO_2_ on water dissociation and hydrogen adsorption/desorption at their interface in an alkaline environment.^[^ ^39^ ^]^ This approach is particularly useful for elucidating the distinct roles of each material by highlighting their contributions within the heterostructure. By optimizing the heterostructure with diverse strategies and periodicity along a single axis, utilizing semivertical heterostructure facilitates catalyst investigation by identifying the active sites where both materials bond and distinguishing the role of each material.

#### 2.1.3 Lateral Heterostructure

Lateral heterostructure involves the horizontal stacking of materials, creating laterally repeated structures. Similar to the semivertical heterostructure, both materials’ surfaces remain exposed, allowing active sites to be accessible. The primary distinction between lateral and semivertical heterostructure lies in the orientation of the stacking, which affects the nature of the material interactions. Unlike semivertical heterostructure, where interactions may be confined to specific regions of overlying materials, lateral heterostructure design ensures that materials interact fully along their interfaces. This is particularly advantageous in systems where materials have comparable thickness, whether in 2D or 3D configurations. Furthermore, when periodic boundary conditions are applied to lateral heterostructures, they allow the exploration of edge sites from both materials in 2D structures. In contrast, for vertical heterostructures, the continuous distinct layers make it difficult to expose the edge sites. As a result, it becomes harder to examine edge‐specific properties in vertical heterostructures, whereas lateral heterostructures provide better access to all edge sites for analysis. This flexibility enables the analysis of systems while ensuring all edge sites of the materials remain accessible for evaluation.

Lateral heterostructure has been employed to pinpoint the roles of each material by exposing both materials as active sites. For alkaline HER electrocatalysts, integrating metal oxides and metals is an effective strategy to enhance water dissociation and hydrogen adsorption/desorption capabilities. Zhu et al. applied lateral heterostructure to simulate Pt single atom supported on Ru/RuO_2_. In this structure, Pt, Ru, and RuO_2_ sites were distinguished and analyzed by arranging Ru and RuO_2_ laterally to expose both materials on the surface. This approach aided in the identification of appropriate materials for distinct roles.^[^ ^40^ ^]^ Furthermore, combining lateral and semivertical heterostructures has proven effective in considering multiple materials and clarifying the effects of each material. For instance, a study analyzed the impact of the Ru_2_P/WO_3_ lateral interface on *N*‐ and *P*‐codoped carbon as an alkaline HER catalyst. This approach utilized Ru_2_P, known for enhancing electrical conductivity through carbon materials, and WO_3_, which was introduced to facilitate water adsorption. The modeling utilized the lateral heterostructure of Ru_2_P and WO_3_ with a vertical construction for the carbon material. This approach revealed how *N* and *P* codoping in carbon improved the electronic conductivity of Ru_2_P/WO_3_. It also disclosed the roles of WO_3_ in water adsorption and dissociation, and Ru_2_P in hydrogen desorption, demonstrating the effectiveness of the lateral heterostructure.^[^ ^41^ ^]^ Similarly, Kumar et al. utilized an approach integrating the lateral heterostructure of NiP_2_ and FeP_2_ with the semivertical heterostructure on Cu(111) for alkaline HER. Specifically, to construct the interface of NiP_2_ and FeP_2_ laterally, they used five layers of NiP_2_(210) and FeP_2_(101), minimizing the interfacial strain. To account for the Cu nanosheet, the NiP_2_/FeP_2_ heterostructure was semivertically bound on Cu(111) (Figure 2c).^[^ ^42^ ^]^ Consequently, utilizing combinations of different heterostructure types enables researchers to adapt their methodologies to specific research objectives and achieve a more comprehensive understanding.

Lateral heterostructure keeps both materials exposed, yet researchers can still focus on the reaction mechanisms of a specific active site when it is clearly identified. For example, a catalyst composed of NiFe selenide and NiFe(OH)_*x*_ was suggested as a crystalline–amorphous heterostructure. This catalyst was synthesized by electrodepositing an amorphous NiFe(OH)_*x*_ shell onto a crystalline selenide core. The computational lateral heterostructure was examined to understand how the presence of selenide influenced the activity of NiFeOOH. While core–shell catalysts were previously introduced with the vertical heterostructure, this system was implemented using lateral heterostructure and specifically focused on the activity of NiFeOOH.^[^ ^43^ ^]^ In addition, lateral heterostructure can also be applied to simulate lamellar structures. One example involves a catalyst consisting of CoFe molten alloy and CeO_2−*x*_N_*x*_, which underwent surface reconstruction during electrochemical reaction. To model the transformation of CoFe into CoFeOOH and the formation of an amorphous phase, ab initio MD (AIMD) was employed. This study further evaluated the effect of nitrogen doping in CeO_2_ by comparing the structures of CoFeOOH, CoFeOOH/CeO_2_, and CoFeOOH/CeO_2−*x*_N_*x*_. They investigated Co within CoFeOOH as the active site for OER mechanism.^[^ ^44^ ^]^ In another example, Ag nanoparticles were deposited on a Ni_3_S_2_ nanosheet, leading to the formation of amorphous NiOOH. The model, which laterally bonded NiOOH@Ag/Ni_3_S_2_, revealed the improved OER activity at the edge site of NiOOH due to Ag nanoparticles (Figure 2d).^[^ ^45^ ^]^ Additionally, lateral heterostructure facilitated the design of FeS_2_/Fe‐doped Ni_3_S_2_ heterostructure formed through F^−^ ion engineering and Fe doping. This study highlighted the importance of lattice‐matching growth, with a comparative analysis of the OER and HER activity of FeS_2_, Ni_3_S_2_, and the FeS_2_/Ni_3_S_2_ heterostructure demonstrating synergistic effects at the adsorption sites near the interface.^[^ ^46^ ^]^ Therefore, these studies have presented how lateral heterostructures can be employed to analyze activity changes when the specific active site forms a heterostructure. Although experimental morphologies appear similar, computational heterostructure types can be selected based on periodic boundary conditions and active sites.

Next, in lateral heterostructures composed of 2D materials, direct covalent bonding between the materials is possible, effectively impacting electronic structures. Hu et al. compared the lateral heterostructures of graphene and hBN, focusing on C—B interface and C—N interface. They found that graphene served as a more effective HER active site compared to hBN in the heterostructures. Specifically, the carbon atom at the C—B interface exhibited the highest HER activity, outperforming both individual graphene and hBN.^[^ ^47^ ^]^ In addition, lateral heterostructure can be applied to vdW heterostructures. This strategy is particularly advantageous for analyzing edge sites of both 2D materials. Yu et al. utilized this strategy to analyze the increased interspacing of MoS_2_ in MoS_2_/graphene vdW heterostructure, induced by the introduction of graphene. This introduction of graphene significantly enhanced the advantages of MoS_2_'s single‐layer properties, improving electrical conductivity. Moreover, this approach allowed them to examine the activity of the edge site, which is known for its effectiveness as an active site with high conductivity.^[^ ^48^ ^]^ Lateral heterostructure has also been used to examine heterostructures formed through surface reconstruction. In a study of Ru‐doped NiFe_2_O_4_ on a NiMoO_4_ nanowire, researchers have investigated the effects of Ru doping on the NiFeOOH/NiOOH heterostructure. They arranged the oxyhydroxide laterally, exposing the edge sites on the surface and calculating the OER activity.^[^ ^49^ ^]^ In summary, lateral heterostructure is a versatile approach, as evidenced by its applications in various electrocatalyst configurations. When combined with semivertical heterostructure modeling, it has shown to be applicable across a range of configurations. These examples also highlight that even for similarly characterized electrocatalysts, including core–shell structures, surface reconstructions, and vdW heterostructures, the construction of computational structures can depend on the specific active sites and research objectives. The relative orientation of the vacuum and surface is particularly important when analyzing edge site activity. These insights into the computational strategies of structure construction provide a foundation for the systematic integration of materials into heterostructured electrocatalyst research.

### 2.2 Lattice Strain Optimization

Computational simulations typically employ periodic boundary conditions to efficiently mimic the infinite nature of real systems in computational finite systems. However, the integration of materials with different lattice constants inherently leads to lattice mismatch, which can significantly influence the catalytic performance of the system.^[^ ^17^ ^]^ This mismatch creates strain that can alter the structural and electronic properties of the materials.^[^ ^50^ ^]^ Additionally, this mismatch forces the materials to either stretch (tensile strain) or compress (compressive strain), creating artificial strains that must be optimized to prevent distorted predictions of material behavior. If lattice strain is not properly managed, the simulated properties, such as bond lengths, adsorption energies, and catalytic activity, may be inaccurately represented, leading to overestimated or underestimated results compared to real experimental data. Therefore, to accurately simulate experimental conditions, it is essential to carefully optimize the strain introduced by computational modeling with periodic boundary conditions.^[^ ^51^ ^]^ In addition, optimizing the lattice strain in computational models is crucial for ensuring that the simulation accurately reflects the corresponding experimental conditions.

Lattice strain is defined as the ratio of the change in length to the original length of a material. In a heterostructure consisting of materials A and B, each material is compressed or relaxed to match the lattice constant of the unit cell, that of optimized heterostructure. The lattice strain of material A is calculated by the formula

(1) $$\varepsilon_{A}=\frac{a_{\text{AB}}-a_{A}}{a_{A}}\times 100\%$$

where $\varepsilon_{A}$ is the lattice strain on material A, $a_{A}$ is the lattice constant of material A, and $a_{\text{AB}}$ is the lattice constant of the optimized heterostructure. The same equation applies to material B. If the lattice strain is positive, tensile strain occurs; if it is negative, compressive strain occurs. In some contexts, tensile and compressive strain are described without explicitly stating positive or negative signs.

For modeling appropriate lattice strain through computational methods, efforts are being undertaken to accurately replicate experimental conditions without distorting the materials’ properties. Zhou et al. proposed a bifunctional catalyst for ORR and HER by modeling the heterostructure using MXene and *N*‐doped graphene with high electrical conductivity, optimizing each material to achieve a lattice strain smaller than 2.12%. They confirmed that the 1.81% tensile strain and 2.12% compressive strain on the graphitic layer were negligible in terms of binding energy and catalytic activity.^[^ ^52^ ^]^ Liu et al. introduced a core–shell OER catalyst utilizing amorphous NiFe oxides and crystalline (Ni,Fe) selenides to enhance the bimetallic effect and the electrical conductivity of oxyhydroxide. They modeled a heterostructure considering that NiFe oxide and hydroxide transform into NiFeOOH during electrochemical reactions. The (110) facet of NiFeOOH was used to minimize interfacial mismatch with (Ni,Fe)Se_2_(100), showing a small lattice strain of 0.54%.^[^ ^43^ ^]^ Additionally, Wang et al. synthesized an alkaline OER catalyst by decorating Ni foam (NF) with Ag nanoparticles and constructing a heterostructure with Ni_3_S_2_. To analyze the Ag/Ni_3_S_2_/NF catalyst, they modeled the heterostructure with NiOOH, which was activated during the operating condition. The presence of NiOOH was confirmed through high‐resolution transmission electron microscopy (HRTEM) and *in* *situ* Raman spectroscopy. They determined each facet of the heterostructure as NiOOH(001), Ag(111), and Ni_3_S_2_(001) to minimize lattice strain (Figure 2d).^[^ ^45^ ^]^ Finally, Hu et al. modeled a lateral heterostructure using the 2D materials hBN and graphene with covalent bonding, proposing its potential as a HER catalyst. The precise matching of lattice constants was essential for forming this heterostructure. hBN and graphene exhibit only a 1.7% lattice mismatch, making them suitable candidates.^[^ ^47^ ^]^ In numerous heterostructure studies, minimizing lattice mismatch between materials has been repeatedly emphasized as crucial for accurately predicting catalytic behavior and performance. Using accurate modeling techniques and quantifying lattice mismatch, researchers have been able to replicate results closely, demonstrating the importance of well‐optimized lattice strain in achieving reliable simulations.

Optimizing lattice strain in computational models is also critical for accurately predicting synthesis feasibility, particularly in epitaxial growth.^[^ ^53^ ^]^ When forming a heterostructure using the epitaxial growth method, it is essential to have nearly identical lattice constants.^[^ ^54^ ^]^ Calculating lattice strain helps evaluate the feasibility of experimental synthesis. For example, Diao et al. modeled a W_2_N/WC heterostructure with 0.59% interfacial strain for trifunctional electrocatalysis (ORR, OER, and HER). In an alkaline environment, WC exhibits Pt‐like characteristics for HER, while W_2_N is effective for OER and ORR. With identical lattice constants of *a* = *b* = 2.238 Å for W_2_N(111) and WC(100), the strain is negligible when WC grows along the [111] direction of W_2_N (Figure 2e). Results confirmed that the synthesized catalyst matched computational predictions, forming the same facets.^[^ ^55^ ^]^ Moreover, Liang et al. suggested that epitaxial growth of pristine NiPS_3_ could improve catalytic activity by forming a heterostructure with Ni_2_P. Although NiPS_3_ alone was not suitable for HER, its combination with Ni_2_P showed promising electrocatalytic potential. The hexagonal Ni_2_P(001) with *a* = *b* = 5.81 Å matches well with the NiPS_3_(001) facet with *a* = *b* = 5.85 Å, resulting in a lattice strain of 0.69% lattice strain. This proved the feasibility of epitaxial growth of Ni_2_P on NiPS_3_.^[^ ^56^ ^]^ These studies underscore the importance of predicting strain effects in heterostructures through calculations and present the potential for experimental synthesis. Additionally, lateral epitaxial growth requires matching lattice constants to minimize the interfacial energy.^[^ ^57^ ^]^ As demonstrated in these studies, the accurate prediction and optimization of lattice strain are the key to bridging computational models with experimental realities. Optimizing lattice strain not only stabilizes interfacial energy but also aids in assessing synthesizability.

## 3 Structural Stability Analysis

### 3.1 Interfacial Binding Energy

Interfacial binding energy is a significant parameter in the design and optimization of heterostructures, providing insights into the stability of the resulting heterostructure. This energy quantifies the interaction between different materials at their interface and can be calculated using the equation.

(2) $$E_{b}=E_{A/B}-(E_{A}+E_{B})$$

where *E*_b_ is the binding energy, *E*_A/B_ represents the total energy of the heterostructure, and *E*_A_ and *E*_B_ are the total energies of the isolated materials A and B, respectively. A more negative *E*_b_ indicates a thermodynamically favorable interaction, suggesting that the formation of the heterostructure is energetically preferable.

Understanding interfacial binding energy is essential for evaluating the structural integrity and the stability of heterostructured catalysts. Strong interfacial interactions with more negative binding energies enhance the stability of the heterostructures under operational conditions. These interactions can lead to charge redistribution, potentially creating unique active sites for electrocatalysis. This can influence catalytic activity by altering the adsorption energies of reactants and intermediates, thereby optimizing reaction kinetics and overall catalytic efficiency. Accurate computation of interfacial binding energy requires careful consideration of exchange‐correlation functionals, atomic precision, and vdW interactions. By tailoring materials and engineering interfaces to optimize binding energy, researchers can develop catalysts with improved stability, activity, and selectivity for applications in energy conversion, environmental remediation, and chemical synthesis.

**Figure 3:**
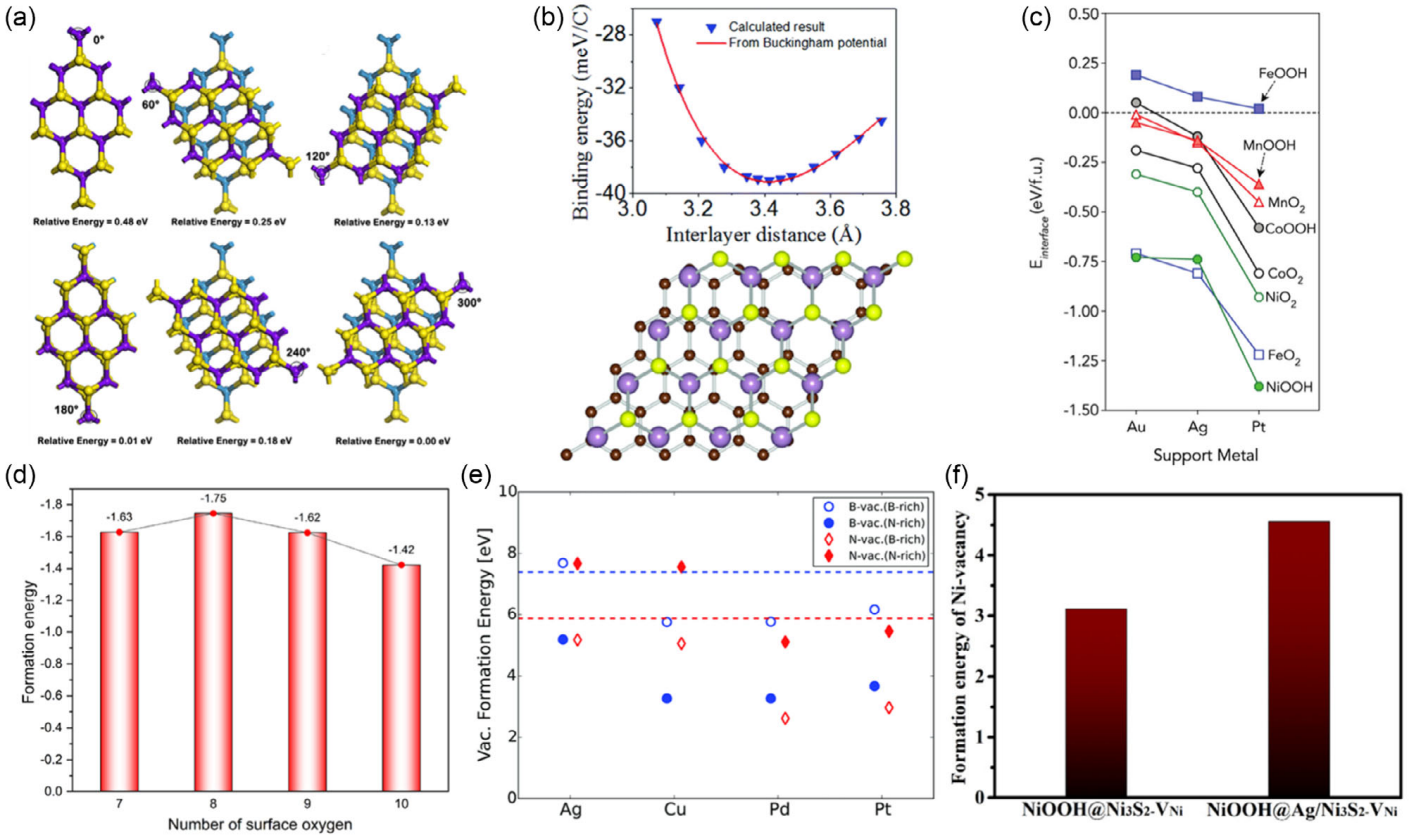
a) Interfacial binding energy based on the angle between 2D TMDs. Reproduced with permission.^[^ ^58^ ^]^ Copyright 2020, American Chemical Society. b) Normalized interfacial binding energy of graphene/MoS_2_ dependent on the number of carbon atoms in graphene. Reproduced under the terms of Creative Commons CC BY‐NC 3.0 license.^[^ ^60^ ^]^ c) Normalized interfacial binding energy between MO_2_, MOOH, and noble metal depending on the number of oxide units. Reproduced with permission.^[^ ^34^ ^]^ Copyright 2019, American Chemical Society. d) Formation energy normalized by the number of added oxygen atoms. Reproduced under the terms of the Creative Commons CC BY license.^[^ ^63^ ^]^ e) Vacancy formation energy of anions *B* and *N* in hBN/Metal heterostructure. The dashed line represents the standalone hBN surface. Reproduced under the terms of Creative Commons CC BY‐NC 3.0 license.^[^ ^21^ ^]^ f) Vacancy formation energy of metal Ni and bond length in NiOOH@Ni_3_S_2_ and NiOOH@Ag/Ni_3_S_2_. Reproduced with permission.^[^ ^45^ ^]^ Copyright 2022, American Chemical Society.

Interfacial binding energy functions as a benchmark for assessing stability, stacking configuration, and bonding states of heterostructured materials. Ge et al. extensively studied 2D dichalcogenide heterojunctions composed of MX_2_ (M = Mo, W; X = S, Se, Te) and revealed how binding energy varies with rotational angles. They found that the MoTe_2_/WTe_2_ heterojunction had the most stable interfacial binding energy of −4.46 eV at a 300° rotational angle, while the MoS_2_/WS_2_ heterojunction showed the weakest interfacial binding energy of −1.77 eV without rotation. This study concluded that eight types of heterostructures generally exhibited stable binding energy at a 180° rotational angle, where interactions occur between metal and nonmetal elements in each layer. A strong correlation between binding energy and layer spacing was observed. The layer spacing tended to decrease at rotational angles greater than 180° before returning to its original state. This analysis highlights the importance of optimizing stacking angles to achieve stable binding energy in the modeling of 2D material‐based heterostructures (**Figure** **3**a).^[^ ^58^ ^]^ Santos et al. compared the binding energies associated with different terminations of a support material in a heterostructure. In the MoS_2_(100)/InAs(111) system, they found that the In‐terminated support was 5.574 eV more stable than the As‐terminated one. The study explained that In atoms tend to move closer to the MoS_2_ layer in the process of relaxation, resulting in a shorter average distance and stronger interaction compared to As—S interaction. Consequently, they reported that the MoS_2_ film interacts more strongly when the support has an In termination.^[^ ^59^ ^]^ Based on these studies, it is evident that interfacial binding energy is influenced by several factors, including interlayer spacing, rotational angles between layers, and terminations of materials. Therefore, by identifying thermodynamically stable configurations, researchers can simulate optimal structures for enhancing catalytic stability and investigating unique interfacial properties.

### 3.2 Normalized Interfacial Binding Energy

When evaluating interfacial binding energy across different systems, normalization becomes important to account for variations in model sizes and interacting surface areas. As the size of the model increases, the binding energy may also increase due to larger interacting areas. Hence, normalization is necessary to accurately compare interfacial interactions. This can be achieved by standardizing the binding energy based on the number of specific atoms or compound units in the system, as described by the following equation.

(3) $$E_{\text{normalized b}}=\{E_{A/B}-(E_{A}+E_{B})\}/N_{i}$$

where *E*_normalized b_ represents the normalized binding energy, *E*_A/B_ is the total energy of the heterostructure, *N*_*i*_ is the number of referenced atoms or compound units, and *E*_A_ and *E*_B_ are the total energies of the isolated materials A and B, respectively.

In heterostructures involving carbon‐based materials such as graphene, the interfacial binding energy is commonly normalized by the number of carbon atoms due to the uniform structure and single‐element composition. Fang et al. optimized a graphene/MoS_2_ heterostructure using the Buckingham potential, which relates the interlayer spacing of the top sulfur atom in MoS_2_ to graphene with the binding energy. They demonstrated a normalized interfacial binding energy of −39 meV C^−1^ atom with a 3.414 Å gap between MoS_2_ and graphene (Figure 3b). This optimization is crucial for stable normalized interfacial binding energy in vdW heterostructures. Furthermore, they observed that positive biaxial strain (tensile strain) weakened the interaction between MoS_2_ and graphene by increasing the spacing. This study highlights the importance of minimizing and optimizing lattice strain to achieve stable normalized interfacial binding energy.^[^ ^60^ ^]^ Similarly, Zhou et al. investigated the binding energy between *N*‐doped graphene and various MXenes in vertical heterostructures. They reported interlayer displacements of 2.08 to 2.40 Å and binding energies of −0.42 eV C^−1^ atom for *N*‐doped graphene on Ti_2_C, −0.32 eV C^−1^ atom for *N*‐doped graphene on V_2_C, −0.25 eV C^−1^ atom for *N*‐doped graphene on Nb_2_C, and −0.23 eV C^−1^ atom for *N*‐doped graphene on Mo_2_C. These results confirmed strong interfacial interactions and significant charge transfers. Therefore, optimizing interlayer distances and normalized interfacial binding energy is essential for accurate modeling and understanding of electronic structures.^[^ ^52^ ^]^ Additionally, *N*‐doped graphene with metallic surfaces of Co(111) and Fe(110) exhibited normalized interfacial binding energies of −0.10 and −0.16 eV C^−1^ atom, respectively.^[^ ^61^ ^]^ Comparing the normalized interfacial binding energy of *N*‐doped graphene with MXenes and metal supports helps identify more stable heterostructures. This method also allows for effective comparisons across different systems, even when model sizes vary depending on the support materials during lattice strain optimization. The previous examples focused on carbon‐based materials, such as graphene. On the other hand, modeling heterostructures involving compounds also utilizes this method for evaluating stability. Back et al. compared the interaction between (oxy‐hydro)oxide nanoribbons and face‐centered cubic (FCC) metals by normalizing the binding energy with respect to the number of (oxy‐hydro)oxide units (Figure 3c). They found that Pt exhibited the most stable normalized interfacial binding energy, followed by Ag and Au. This stability trend is explained by the increasing oxophilicity of the support metals and the proximity of their *d*‐bands to the Fermi level, moving from Au to Pt.^[^ ^34^ ^]^ This approach demonstrates the effectiveness of normalization for comparing different systems, even when model sizes and compositions vary.

### 3.3 Formation Energy

In the previous section, we introduced (normalized) interfacial binding energy to understand the bonding interactions between pristine materials. This section focuses on formation energy, which is essential for evaluating surface and composition stability in heterostructures. While formation energy encompasses a broad range of applications, we specifically focus on cases where components are added or substituted. Formation energy is a fundamental concept in understanding the thermodynamic stability of heterostructured catalysts, providing insights into surface stability. Accurately determining the formation energy requires the establishment of model‐specific equations that account for the total number of components involved. This energy is calculated using the equation.

(4) $$E_{f}=(E_{\text{total}}-E_{A/B}-\sum_{i}n_{i}\mu_{i})/N_{i}$$

where *E*_f_ represents formation energy, *E*_total_ is the total energy of the heterostructure with added or substituted constituent units, *E*_A/B_ is the total energy of the original heterostructure, *n*_*i*_ represents the number of constituent unit, *μ*_*i*_ is the chemical potential of constituent unit *i*, and *N*_*i*_ is the total number of added or substituted constituent units. A negative formation energy indicates a thermodynamically stable state compared to its original states, which supports favorable surface stability. Additionally, normalizing the formation energy by the number of referenced atoms or compounds enables the comparison of different surface conditions. The following examples illustrate the application of formation energy calculations to optimize metal oxide compositions and the quantity of adsorbed oxygen atoms. First, a study was conducted to investigate the origins of the high performance of Ag and MnO_*x*_‐based catalysts for ORR. The ratio of Ag to MnO_*x*_ and the composition of MnO_*x*_ both significantly influence catalytic activity, necessitating appropriate modeling strategies. To precisely simulate Ag–MnO_*x*_ catalysts in DFT modeling, various MnO_*x*_ configurations were investigated based on experimental observations. In the case of MnO_2_, formation energy was corrected by the oxygen chemical potential and normalized to the number of Mn atoms. The study revealed that the Mn_18_O_36_ nanostripe@Ag(111) had a formation energy of −0.08 eV Mn^−1^ atom, while the Mn_7_O_9_ nanoisland had a formation energy of −0.75 eV Mn^−1^ atom.^[^ ^62^ ^]^ Therefore, they effectively utilized formation energies to assess the model structures by considering the configuration and composition of MnO_*x*_. In another study, Liu et al. synthesized an Ir nanorod–MoO_3_ catalyst embedded in a graphitic carbon layer using various semiconducting metal oxides through the electrospinning method, with IrO_2_ being reduced. To model the heterostructure based on HRTEM images, they utilized the MoO_3_(040) and Ir(111) facets. To verify that the electron‐poor Ir state promotes the OER, they compared the OER activity of an oxygen‐covered Ir metallic surface. The formation energy for eight additional surface oxygen atoms was −1.74 eV O^−1^ atom, compared to −1.63 eV O^−1^ atom for seven oxygen atoms, −1.62 eV O^−1^ atom for nine oxygen atoms, and −1.42 eV O^−1^ atom for ten oxygen atoms (Figure 3d). The model with eight additional surface oxygen atoms, being the most stable in terms of formation energy, was compared with the OER‐active model with seven oxygen atoms and metallic Ir on MoO_3_. As a result, it was revealed that the added oxygen atoms act as proton acceptors and improve OER activity.^[^ ^63^ ^]^ In this way, formation energy functions not only as an indicator for assessing the stability of models but also as a criterion for considering oxidation states and additional active sites, which are important factors in catalytic reaction environments.

Next, the formation energy can be calculated to analyze the stability of substituting specific elements within a heterostructure. In the study of *N*‐doped graphene (NG) supported on Fe_3_C as an ORR catalyst, stability was explored by substituting different metals into the Fe positions in the first and second top layers. This analysis involved comparing the energy of NG/Fe_3_C after the removal of all Fe atoms in the first layer with another metal, with corrections made for the number of substituted metal atoms and the preferred metal carbide units. When substituting Fe in the first layer with another metal, the referenced constituent unit was pure metal. The formation energy was then calculated by comparing it with a model where all Fe atoms were removed from the first layer and adjusting for the substituted metal atoms. According to this equation, a negative formation energy indicates that Fe is more likely to be replaced by other metals in NG/Fe_3_C. Additionally, for some metals that prefer to exist as carbides rather than in their pure metal when carbon is present, metal carbides were used as the referenced constituent unit. This adjustment accounted for the tendency of the substituted metal to form a separate carbide phase in the presence of graphite. By establishing these equations, the substitutions of Fe by metals and metal carbides in Fe_3_C encapsulated by NG were effectively modeled. Furthermore, for metals like Ni or Co, which prefer to exist in the metal carbide phase, formation energies were calculated across various substitution concentrations, providing insights into the distribution of metal layers and the concentrations of stable models.^[^ ^64^ ^]^ Therefore, by computationally using formation energy to reflect the stable components of the catalyst and its environment in heterostructures, one can propose and compare metal distribution, optimal concentrations with encapsulation, and other key factors for enhancing catalytic performance.

### 3.4 Vacancy Formation Energy

Vacancy formation energy represents the energy required to create a vacancy within the crystal lattice of a material. It is a critical parameter for understanding the stability of heterostructures and for modeling defective surface conditions. When vacancy formation is energetically favored, vacancies can become reactive species, indicating the potential involvement of lattice atoms in catalytic reactions. This understanding is crucial for understanding the structural integrity and performance of heterostructured catalysts, as vacancies can significantly influence catalytic activity by modifying surface properties and reaction kinetics. By accurately calculating vacancy formation energies in computational modeling, researchers can design heterostructured catalysts that are optimized for enhanced stability and catalytic performance for various applications. The vacancy formation energy (*E*_vac_) can be calculated using the equation.

(5) $$E_{\text{vac}}=E_{\text{vacant}}-E_{\text{pristine}}+\sum_{i}n_{i}\mu_{\text{vac}}$$

where *E*_vac_ represents the vacancy formation energy, *E*_vacant_ is the total energy of the defective structure with the created vacancy, and *E*_pristine_ is the total energy of the pristine structure without any vacancies. *μ*_vac_ represents the chemical potential of the vacancy atom, and *n*_*i*_ is the number of vacancy atoms. In certain cases, an energy term considering the Fermi level is included, especially for semiconductors; however, it is often disregarded when comparing neutral structures.^[^ ^59^ ^]^ A lower vacancy formation energy indicates a more favorable vacancy formation. This metric is essential for determining the stability of heterostructures and the activity of lattice vacancy sites, ultimately aiding in the design of more efficient and stable catalysts.

Through the comparison of vacancy formation energy, one can analyze whether a heterostructure promotes or restricts vacancy formation. Back et al. reported that in hBN/metal‐support heterostructured catalysts, vacancies in hBN are required to activate ORR activity. To determine the preference for B and N vacancy formation in hBN on Ag, Pd, Pt, and Cu supports, they calculated the corresponding vacancy formation energies. Compared to vacancy formation in isolated hBN, B vacancy formation is generally preferred in the presence of a metal support, except under B‐rich conditions on Ag. Notably, B vacancies are most stably formed on Cu support (Figure 3e), highlighting the crucial role of the support in facilitating vacancy formation.^[^ ^21^ ^]^ Santos et al. also analyzed vacancy formation energy in a heterostructure formed by 3D InAs(111) with As and In terminations and 2D MoS_2_(100). They examined the formation energies of As, In, and S vacancies at various sites, including corner, edge, and center for different bonding states. Their calculations revealed that vacancy formation is more favorable at the interface interacting with MoS_2_ than within the bulk for both As and In terminations. In particular, In vacancies in the In termination had negative vacancy formation energy, indicating a highly favorable spontaneous formation, in contrast to As vacancies. S vacancies in MoS_2_ at the interface exhibited high positive vacancy formation energy, suggesting a low likelihood of occurrence. Despite the positive vacancy formation energy for As, its proximity to zero suggests that As vacancies could still form under thermal and experimental conditions. This study underscores that vacancies in InAs/MoS_2_ heterostructures are more likely to form at the surface interface rather than in the bulk of InAs.^[^ ^59^ ^]^ It also suggests that it is crucial to consider various factors, such as bonding states, interface interactions, bulk or surface sites, and the specific material characteristics, to comprehensively investigate vacancy formation energy. Furthermore, vacancy formation energy in heterostructures can be used as an indicator of structural stability. Generally, the formation of vacancies can alter material properties or increase the concentration of active species, thereby enhancing catalytic activity. However, if spontaneous defect formation is energetically favored, the overall stability of the catalyst may decrease. Experimentally, a higher concentration of dissolved metals from the catalyst into the electrolyte can indicate reduced stability.

Similarly, in computational studies, metal vacancy formation energy is often used to compare the stability of different catalysts. For instance, Wang et al. addressed stability issues in Ni_3_S_2_ utilizing a heterostructure with noble metal Ag. They determined the improved stability by calculating the Ni vacancy formation energy for NiOOH@Ag/Ni_3_S_2_ and NiOOH/Ni_3_S_2_. They found that NiOOH@Ag/Ni_3_S_2_ required 4.55 eV for Ni vacancy formation, compared to 3.11 eV for NiOOH/Ni_3_S_2_, indicating that Ni vacancies are more difficult to form in NiOOH@Ag/Ni_3_S_2_ (Figure 3f). This higher‐energy requirement was attributed to the shorter Ni—O bond length in NiOOH@Ag/Ni_3_S_2_, which suggests stronger Ni—O bonding, contributing to increased stability and potentially reducing Ni dissolution in the electrolyte.^[^ ^45^ ^]^ Vacancy formation energy also reflects the stability of defective surfaces. Reda et al. calculated the vacancy formation energy associated with the removal of 1–4 carbon atoms at the Fe_3_C(010)/*N*‐doped graphene(NG) interface. They found that while the vacancy formation energy for removing three carbon atoms was positive, the energy was sufficiently small (12 meV C^−1^ atom), indicating that these atoms could still be removed under pyrolytic conditions. Consequently, the thermodynamically most stable state with the removal of all four carbon atoms was used in their ORR analysis.^[^ ^65^ ^]^ Therefore, vacancy formation energy can be utilized both to simulate catalyst surfaces by introducing defects in heterostructures and to assess the overall stability of the heterostructured catalysts.

## 4 Electronic Structure Analysis

### 4.1 Charge Density Difference

Charge density difference (CDD) analysis visualizes and quantifies the redistribution of electron density upon the formation of heterostructures. This analysis is essential for understanding the electronic interactions at the interface of two materials. Charge density redistribution analysis, particularly Bader charge analysis, focuses on the spatial distribution of electronic charge within a material. It decomposes the total charge density into contributions from individual atoms, providing a microscopic view of charge distribution.^[^ ^66^ ^]^

Bader analysis, a quantum theory‐based method, divides electronic charge density into atomic contributions by identifying atomic regions with minimum charge density, enabling the assignment of charge to atoms in molecules and condensed phase systems. This method complements CDD analysis by offering an atomistic quantification of charge distribution. The resulting quantitative insights enable the calculation of charge transfer, vital for predicting catalytic activity and stability in heterostructured catalysts.^[^ ^66^, ^67^ ^]^

More specifically, charge density can be described as a gradient, moving from a grid point (*i, j, k*) along the direction which maximizes itself. Charge density gradient can be calculated.

(6) $$\nabla \rho (i,j,k)\cdot \hat{r}(di,dj,dk)=\frac{\Delta \rho }{\left|\Delta \hat{r}\right|}$$

where *di, dj, dk* are each assigned the values {−1, 0, 1}, but excluding *di = dj = dk* = 0. The change in charge density

(7) Δρ=ρ(i+di,j+dj,k+dk)−ρ(i,j,k)

and the distance

(8) $$\left|\Delta \vec{r}\right|=\left|\vec{r}\left(i+di,j+dj,k+dk\right)-\left|\vec{r}\left(i,j,k\right)\right|\right|$$

are evaluated between neighboring points. $\vec{r}\left(i,j,k\right)$ is the Cartesian vector to the grid point (*i, j, k*). The steepest ascent step, $\vec{r}\left(di,dj,dk\right)$, is known to maximize the positive value for ∇ρ(i,j,k). If no such point exists, the point (*i, j, k*) is the charge density maximum. Based on the trajectories toward the charge density maximum points, Bader volume can be designated, and the charge density within each Bader region is used to quantify the charge density.

Furthermore, the CDD of a heterostructure model can be calculated using the following equation.

(9) $$\Delta \rho =\rho_{A/B}-\rho_{A}-\rho_{B}$$

where Δρ represents the CDD of the heterostructure, $\rho_{A/B}$ is the charge density of the heterostructure, $\rho_{A}$ and $\rho_{B}$ are the charge density of the isolated materials A and B, respectively.^[^ ^68^ ^]^ Researchers employ the CCD analysis, including Bader charge analysis, to validate heterostructure models by quantitatively confirming their characteristics and understanding the electronic interactions at the interface.

### 4.2 Electron Localization Function

Heterostructured catalysts integrate different materials to enhance catalytic performance through synergistic interactions at their interfaces. ELF provides detailed visualizations of electron distribution within materials, highlighting the nature of bonds, electron pairs, and charge transfer processes. By analyzing ELF, researchers can examine electron distribution across interfaces, identifying changes in bonding and electronic structure induced by heterostructure formation. This analysis reveals regions of electron accumulation or depletion, indicating critical charge transfer between the constituent materials. This understanding is vital for optimizing catalyst design and improving catalytic reactions.

The ELF is a dimensionless scalar field that measures the spatial localization of electrons and can further map out electron pair probability for multielectron systems. It ranges from 0 to 1, where a value of 1 indicates perfect localization and 0 indicates complete delocalization. The ELF was originally defined by Becke and Edgecombe in 1990, as shown in the equation below.

(10) $$ELF\left(r\right)=\frac{1}{1+{\left(\frac{D\left(r\right)}{D_{h}\left(r\right)}\right)}^{2}}$$

where *D*(*r*) is the curvature of the electron pair density, and *D*_*h*_(*r*) is the corresponding quantity for a homogeneous electron gas with the same density. The ELF is instrumental in visualizing various types of chemical bonds, including covalent, ionic, and metallic bonds, by illustrating how electrons are redistributed within materials. It can distinguish between lone pairs and bonding pairs of electrons, offering insights into the geometry and reactivity of molecules and materials. In heterogeneous catalysts, ELF analysis is particularly useful for interpreting electron localization around active sites and understanding how these localizations influence catalyst activity.^[^ ^69^ ^]^ This makes ELF a powerful tool for optimizing the design of heterostructured catalysts. Using ELF in the investigation of these catalysts, researchers can gain deeper insights into electronic interactions at the atomic level. This facilitates the strategic design of more efficient catalysts by identifying optimal configurations and compositions that maximize performance. ELF's detailed analysis clarifies the connection between electronic structure and catalytic activity, facilitating the development of advanced materials tailored for specific catalytic applications.

### 4.3 Density of States

Density of states (DOS) is a fundamental concept in solid‐state physics and computational materials science that describes the distribution of electron states relative to the Fermi level within a heterostructure. In the context of heterostructures, DOS plays a crucial role in providing a global perspective on the electronic structures. It is particularly effective for examining heterostructure models via DFT. DOS function, *D*(*E*), describes the number of electronic states available at each energy level *E* within a material. It is defined mathematically as

(11) $$D\left(E\right)=\sum_{i}\delta \left(E-E_{i}\right)$$

where *E*_*i*_ are the energy eigenvalues and δ is the Dirac delta function. DOS is integral to understanding the electronic structure of a material because it indicates how electrons occupy various energy levels and how these distributions are affected by the formation by heterostructures, directly optimizing their electronic properties.^[^ ^70^ ^]^

DOS analysis is pivotal in heterostructures for several reasons. It elucidates how the electronic structure is modified when two materials form a heterostructure, revealing new electronic states at the interface. These changes in the DOS signify the emergence of unique states that can potentially enhance catalytic performance. For example, semiconducting properties of individual materials can shift to metallic properties when forming heterostructures. These new electronic states at the Fermi level can facilitate the charge transfer and increase the electrical conductivity. Moreover, DOS analysis aids in understanding charge transfer dynamics between the materials within the heterostructure. By examining the alignment of energy bands and shifts in the Fermi level before and after heterostructure formation, researchers can track electron redistribution across the interface. Understanding and optimizing these electronic properties through DOS analysis are instrumental in designing efficient and selective heterostructured catalysts.

To accurately determine the DOS, the eigenvalues *E*_*i*_ and eigenfunctions $\psi_{i}\left(r\right)$ for the heterostructure are calculated using the Kohn–Sham equation as seen below.

(12) $$\left[-\frac{\hbar^{2}}{2m}\nabla^{2}+V_{\text{eff}}\left(r\right)\right]\psi_{i}\left(r\right)=E_{i}\psi_{i}\left(r\right)$$

where *V*_eff_(r) is the combination of external potential, Hartree potential, and exchange correlation potential. The energy levels *E*_*i*_ are sampled over the Brillouin zone using a dense *k*‐point mesh and the delta functions are broadened to finite‐width Gaussian or Lorentzian functions for practical numerical evaluation using the below formula.

(13) $$D\left(E\right)=\sum_{i}\frac{1}{\sqrt{\pi }\sigma }e^{-{\left(\frac{E-E_{i}}{\sigma }\right)}^{2}}$$

where *σ* is the broadening parameter. This method yields a smooth DOS curve, which is crucial for analyzing the global perspective of the electronic properties of heterostructure materials.^[^ ^71^ ^]^

Understanding the *d*‐band center is essential for improving catalytic performance in heterostructured catalysts. The influence of the *d*‐band center on catalytic properties was highlighted by Hammer and Nørskov, who demonstrated that its position can predict the reactivity of TMs and suggested it as a descriptor for catalytic activity. The *d*‐band center is a critical factor in determining the binding strength of adsorbates on surfaces. The *d*‐band center, *ε*_*d*_, is calculated as.

(14) $$\varepsilon_{d}=\frac{\int_{-\infty }^{\infty }E\times \rho \left(E\right)dE}{\int_{-\infty }^{\infty }\rho \left(E\right)dE}$$

where *ρ*(E) is the DOS and *E* is the relative energy to the Fermi level. The position of the *d*‐band center affects the binding strength of adsorbates.^[^ ^68^ ^]^ A *d*‐band center closer to the Fermi level typically indicates stronger binding of adsorbates on the catalyst surface, which can enhance catalytic activity by facilitating stronger interaction with reactants and intermediates. However, if the binding is excessively strong, it can lead to catalyst poisoning. Leveraging the concept of the *d*‐band center allows researchers to optimize the electronic structure of heterostructured catalysts, achieving higher activity and selectivity for specific electrochemical reactions. By carefully selecting and combining materials, computational materials scientists can engineer the *d*‐band center to tailor the catalyst's reactivity, guiding the development of efficient and sustainable catalytic systems.

### 4.4 Work Function

Work function is defined as the energy required to remove an electron from a material and elevate it to the vacuum level. In heterostructures composed of different materials, this analysis helps understand how the interface alters surface potential energy and electron emission capability. By assessing the work function of each material and the heterostructured system, researchers can discern shifts in the Fermi level, quantify charge transfer dynamics, and characterize changes in surface dipole moments. These alterations directly impact catalytic activity by influencing the adsorption and desorption of reactants and intermediates on the catalyst surface, thereby guiding the optimization of catalyst design for enhanced performance.^[^ ^72^ ^]^

DFT is useful in determining the work function (Φ) of a heterostructure. After calculating the electrostatic potential across the slab model, the work function is determined from the difference between the vacuum level (*V*_vacuum_) and the Fermi level (*E*_f_).

(15) $$\Phi =V_{\text{vacuum}}-E_{f}$$

This calculation provides valuable insights into how the interface between different materials influences surface potential and charge transfer processes.^[^ ^73^ ^]^

The concept of a Schottky heterojunction is frequently employed when creating high‐performance heterostructured electrocatalysts. A Schottky heterojunction, named after Walter H. Schottky, forms at the interface between two materials, typically a semiconductor and a metal, with different work functions, leading to the formation of a built‐in potential barrier. This barrier, arising from the work function disparity, regulates the transport of charge carriers, electrons or holes, across the junction, resulting in distinct electronic properties and behaviors. This enables precise manipulation of the electronic structure and surface properties to enhance catalytic activity and selectivity for designing heterostructures.^[^ ^74^ ^]^ The Schottky heterojunctions in heterostructured catalysts are also pivotal for catalytic reactions that rely on charge transfer processes in electrocatalysis. The built‐in potential barrier at the heterojunction interface dictates the flow of electrons or holes, enabling efficient charge separation.^[^ ^75^ ^]^ Tailored engineering of surface properties in each constituent material allows for the modulation of reaction kinetics, with one material acting as a cocatalyst for intermediate activation and the other as a promoter to stabilize reaction intermediates.^[^ ^76^ ^]^ Control over band alignment at the interface further influences electron transfer energetics, enhancing reaction efficiency and minimizing energy losses. The unique electronic properties of Schottky heterojunctions provide opportunities for precise tuning of reaction pathways, promoting desired reactions while suppressing undesirable side reactions. By leveraging Schottky heterojunctions and conducting work function calculations, researchers can ensure that heterostructured catalysts are well designed to fulfill their intended purpose. These methodologies provide critical insights into the electronic interactions at interfaces, guiding the development of advanced catalysts tailored for specific catalytic applications.

## 5 Applications of Heterostructured Electrocatalyst

Developing highly efficient heterostructured electrocatalysts hinges on a thorough understanding of the key factors that govern their performance. This section will explore computational approaches on heterostructured catalysts, focusing on specific electrochemical reactions. By investigating the methodologies and activity descriptors in heterostructure research, we aim to present meaningful insights and comprehensive frameworks for interpreting calculation results of heterostructured electrocatalysts. These insights will provide a practical guide for advancing the application of heterostructured electrocatalysts within computational materials science.

### 5.1 Oxygen Evolution Reaction (OER)

The significance of hydrogen energy is increasingly acknowledged for its role in mitigating climate change. Water electrolysis technology is essential for the production of green hydrogen, which generates no carbon dioxide emissions. This process relies on two key reactions: OER and HER. The OER, specifically, poses a significant challenge due to its slow kinetics, which involve a complex four proton‐electron transfer process that limits the overall efficiency of water electrolysis. The acidic OER follows the reaction of $2H_{2}O+*\;⇄\;$*+$O_{2}+4H^{+}+4e^{-}$ and the alkaline OER follows the reaction of $4{\text{OH}}^{-}+*⇄*+O_{2}+2H_{2}O+4e^{-}$. These mechanisms contain the main oxygen intermediates of *OH, *O, and *OOH. To evaluate the OER activity, the Gibbs free energy differences between reaction steps are compared at the equilibrium potential (*U* = 1.23 V). This enables the identification of the potential‐determining step (PDS) and the calculation of the theoretical overpotential required to allow the reaction to occur.^[^ ^77^ ^]^ Superior OER performance is achieved by the balanced distribution of Gibbs free energy among the oxygen intermediates to minimize the theoretical overpotential. Therefore, enhancing OER catalytic efficiency can be accomplished by tuning the charge density distribution and electronic structure of heterostructures.

**Figure 4:**
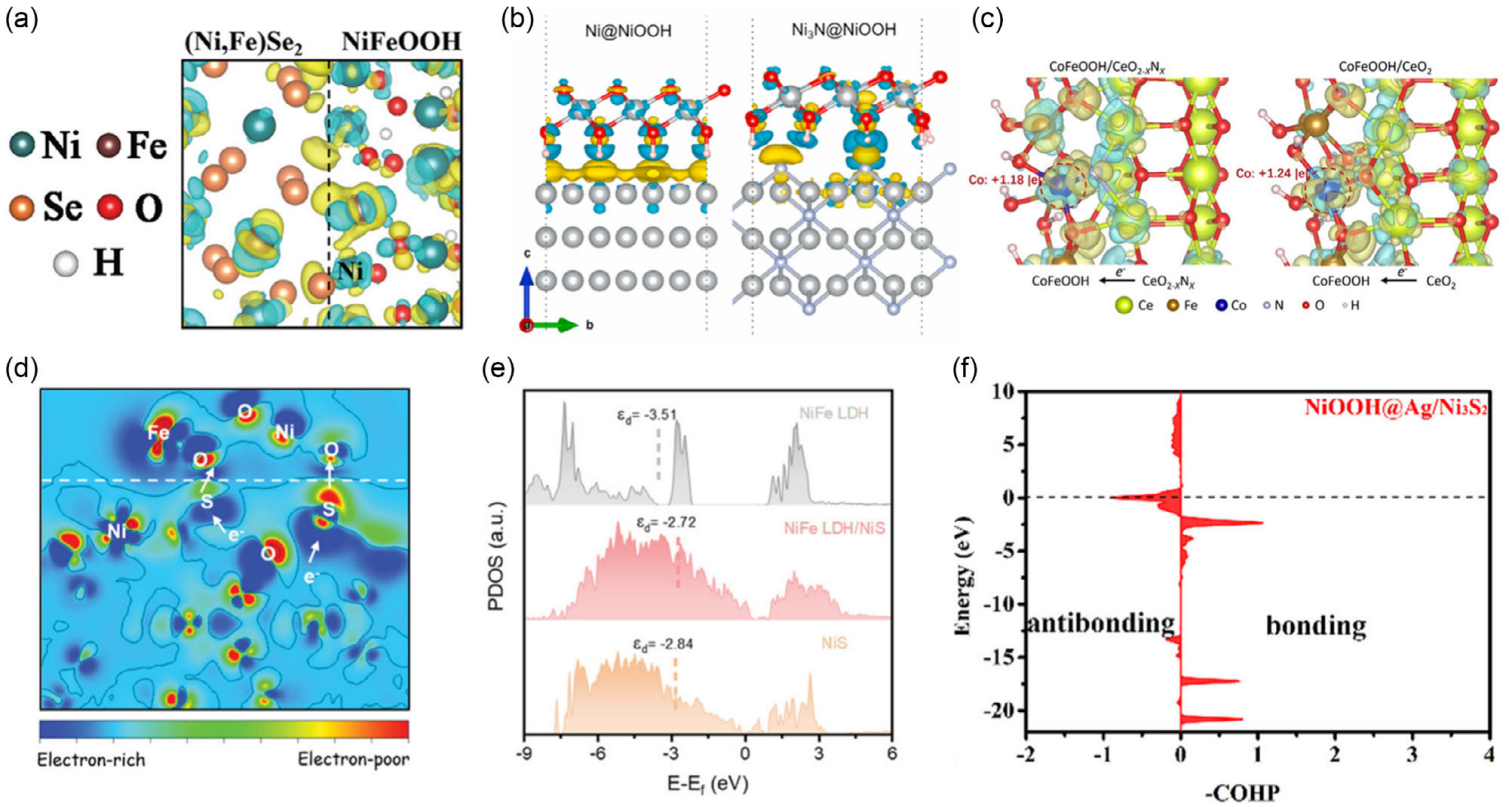
Electronic structure and activity analysis on OER heterostructured catalyst. a) CDD of NiFeOOH/(Ni,Fe)Se_2_. Reproduced with permission.^[^ ^43^ ^]^ Copyright 2021, WILEY‐VCH GmbH. b) CDD of Ni@NiOOH and Ni_3_N@NiOOH. Reproduced with permission.^[^ ^30^ ^]^ Copyright 2020, Elsevier. c) The Bader charge of Co and CCD in CoFeOOH/CeO_2−*x*_N_*x*_ and CoFeOOH/CeO_2_. Reproduced under the terms of the Creative Commons CC BY license.^[^ ^44^ ^]^ d) 2D CCD of NiFe LDH/NiS. e) PDOS of NiFe LDH, NiFe LDH/NiS, and NiS. Reproduced with permission.^[^ ^35^ ^]^ Copyright 2021, WILEY‐VCH GmbH. f) COHP of NiOOH@Ag/Ni_3_S_2_. Reproduced with permission.^[^ ^45^ ^]^ Copyright 2022, American Chemical Society.

CDD analysis provides valuable insights into electron transfer phenomena and identifies critical factors influencing catalytic activity. For example, the charge transfer from LDH or oxyhydroxide to other materials is known to enhance OER performance. Specifically, electron depletion and positive charging facilitate stable adsorption of OER intermediates, thereby reducing the theoretical overpotential associated with the PDS. Liu et al. proposed a core–shell interfacial heterostructure of NiFe LDH with (Ni,Fe)Se_2_, where crystalline selenide compensates for the low conductivity of amorphous LDH. During electrochemical processes, LDH phases transition between M(OH)_2_ and MOOH depending on the applied potential and the presence of intercalated ions. Under oxidizing conditions, NiFe LDH transforms into NiFe oxyhydroxide and the interface between NiFeOOH(110) and (Ni, Fe)Se_2_(100) was modeled. Differential charge density analysis revealed that Ni in NiFeOOH reached a higher oxidation in the heterostructure, with significant charge redistribution and decreased charge density (**Figure** **4**a). This charge reduction in Ni, which acts as the active site, facilitated more stable adsorptions of OER intermediates, leading to reduced theoretical overpotential and improved OER activity.^[^ ^43^ ^]^ Chen et al. suggested a Co_3_S_4_@NiFe LDH core–shell heterostructure designed to enhance stability against sulfide degradation. CDD and Bader charge analysis showed that ≈1.725 electrons were transferred from NiFe LDH to Co_3_S_4_. Gibbs free energy calculations identified the PDS as the *O → *OOH step, with Co_3_S_4_@NiFe LDH exhibiting a lower energy barrier (1.75 eV) compared to NiFe LDH (1.86 eV) and Co_3_S_4_ (2.03 eV). They demonstrated that electron depletion in NiFe LDH contributed to reduced free energy for the PDS.^[^ ^78^ ^]^ Similarly, Ni_3_P/NiFe LDH showed electron transfer from NiFe LDH to Ni_3_P, with NiFe LDH displaying a lower electrostatic potential than Ni_3_P. This indicated that a smaller work function of NiFe LDH made it prone to lose electrons into Ni_3_P.^[^ ^79^ ^]^ Furthermore, electron depletions in NiFe LDH were investigated in Ni_2_Fe LDH/FeNi_2_S_4_/NF, NiSe@NiFe LDH/NF, and Co_9_S_8_@NiFe LDH, demonstrating how electron redistribution stabilizes OH and OOH adsorption energies, thereby mitigating the PDS associated with OOH* formation.^[^ ^80^ ^]^

This charge transfer strategy also can be applied to other material systems. Gao et al. introduced a heterostructured catalyst with a Ni_3_N shell and Ni_3_N/Ni core, confirming the transformation into NiOOH under alkaline conditions using *in* *situ* Raman spectroscopy and XPS. In Ni@NiOOH and Ni_3_N@NiOOH heterostructure models, the electron migration from NiOOH to Ni_3_N particularly contributed to favorable adsorption and desorption capabilities of OER intermediates (Figure 4b). Comparative analysis of Bader charges at active sites provided insights into charge distribution.^[^ ^30^ ^]^ Likewise, Wang et al. investigated an NiOOH@Ag/Ni_3_S_2_ heterostructure, where self‐assembled NiOOH acted as the active site for OER. Bader charge comparison between NiOOH@Ag/Ni_3_S_2_ and NiOOH/Ni_3_S_2_ catalysts revealed that the lower Bader charge of Ni atom in NiOOH@Ag/Ni_3_S_2_ promoted a higher valence state and OER activity with Ag.^[^ ^45^ ^]^ These examples show that when NiOOH loses charge density, the energy required for the OH* formation step is reduced, leading to superior catalytic performance. These findings highlight the importance of CDD analysis in elucidating and optimizing catalyst performance for efficient OER applications.

Conversely, gaining charge at active sites can be advantageous, depending on the specific catalytic mechanism and conditions. Zeng et al. proposed a CoFeOOH/CeO_2−*x*_N_*x*_ heterostructure for alkaline OER. They synthesized FeCo alloy/oxide and Ce hydroxide/oxide laminate composites through a molten alloy cooling and dealloying process, followed by annealing in an NH_3_ environment to produce CoFe/CeO_2−*x*_N_*x*_ and CoFe/CeO_2_ structures. Bader charge analysis of CoFeOOH/CeO_2−*x*_N_*x*_ revealed that nitrogen introduction led to increased electron transfer from CeO_2−*x*_N_*x*_ to CoFeOOH, resulting in Co having the lowest Bader charge value of +1.18 |e| compared to +1.24 |e| for CoFeOOH/CeO_2_ and +1.45 |e| for bare CoFeOOH (Figure 4c). This reduction in Bader charge signifies electron accumulation on Co and weakened binding energies of intermediates, which is beneficial for OER catalysis.^[^ ^44^ ^]^ Moreover, in Schottky heterojunctions, such as the one proposed by Wen et al. involving NiFe LDH and NiS, the result of electron transfer plays a crucial role in enhancing OER activity. This catalyst facilitates electrolyte penetration and bubble diffusion through porous NiS, contributing to improved catalytic performance. A 2D CDD analysis confirmed electron transfer from metallic NiS to semiconducting NiFe LDH, leveraging the smaller work function of NiS compared to NiFe LDH to promote electron distribution toward the LDH (Figure 4d). Gibbs free energy profiles of the OER exhibited that the Schottky junction between NiFe LDH and NiS significantly reduced the theoretical overpotential to 0.46 V, a marked improvement compared to the individual NiFe LDH and NiS catalysts. This result underscores the efficacy of charge rearrangement in enhancing catalytic efficiency through material interaction.^[^ ^35^ ^]^ In other cases, LDHs also gain electrons with higher OER activities, including Ni/NiFe LDH and CoFe LDH‐MXene systems, indicating the versatility of electron transfer mechanisms in catalytic improvements.^[^ ^81^ ^]^ These insights underscore the importance of charge transfer analysis in tailoring catalyst design for efficient electrochemical reactions, such as the OER.

Importantly, the metallic DOS that reduces the bandgap and the higher electron density at the Fermi level in heterostructures are key factors in achieving excellent OER activity. Furthermore, the *d*‐band center provides the electronic structure's effect on stabilization or moderation of oxygen intermediates adsorption energies. For example, the NiFeOOH/(Ni,Fe)Se_2_ heterostructure illustrated metallic properties near the Fermi level in the total DOS (TDOS) analysis, a notable difference from the semiconducting nature of NiFeOOH. In the Gibbs free energy profile, it was observed that the PDS shifted from the OH*→O* step in NiFeOOH to the O*→OOH* step in the heterostructure. This shift reduced the theoretical overpotential to 0.98 V, thus confirming the higher activity of the heterostructure.^[^ ^43^ ^]^ DFT calculations further revealed that coupling NiFe(OH)_2_(001) with NiS(110) significantly modulates the electron density around the Fermi level, resulting in a more conductive electronic structure (Figure 4e). From the projected DOS (PDOS) analysis, the higher *d*‐band center of metals in the NiFe LDH/NiS suggested stronger binding strengths with oxygen intermediates.^[^ ^35^ ^]^ Conversely, in some cases, a lower *d*‐band center is advantageous for reducing the Gibbs free energy of the PDS. For instance, comparisons of CoFeOOH/CeO_2−*x*_N_*x*_, CoFe/CeO_2_, and CoFeOOH revealed that a downshift of the *d*‐band center correlated with improved OER activity. To further alleviate the theoretical overpotential, nitrogen‐doped CeO_2_ was used to achieve moderate adsorption strength, lowering the *d*‐band center of the active Co site.^[^ ^44^ ^]^ On the other hand, the NiOOH@Ag/Ni_3_S_2_ system with improved electrical conductivity and strengthened binding energies of oxygen intermediates contributes to increased OER activity. Notably, in NiOOH@Ag/Ni_3_S_2_, the crystal orbital Hamilton population (COHP) analysis between the Ni atom at the active site and the adsorbed oxygen atom exhibited a decrease in antibonding state filling at energies below the Fermi level in NiOOH@Ag/Ni_3_S_2_, enhancing the interaction with intermediates. Additionally, it exhibited significantly higher electron density at the Fermi level compared to NiOOH/Ni_3_S_2_, reflecting its superior conductivity. Therefore, the combination of higher conductivity, as demonstrated by DOS, and the reduction in antibonding state filling, as shown by projected COHP analysis, suggests superior OER activity for surface reconstruction catalysts (Figure 4f).^[^ ^45^ ^]^ In summary, forming heterostructures allows LDHs, oxyhydroxides, and oxides to manipulate electron densities, stimulate metallic DOS characteristics, and modulate the adsorption energy of OER intermediates. These adjustments augment electrical conductivity and reduce the Gibbs free energy of PDS. Therefore, understanding how the properties of active sites evolve within heterostructures and identifying the key factors that enhance OER activity are essential for advancing heterostructure catalysts.

### 5.2 Hydrogen Evolution Reaction (HER)

HER is crucial for hydrogen energy, a promising next‐generation energy source due to its high energy density. Given its significance, HER is employed at the cathode in conjunction with various effective oxidation reactions, such as OER and UOR, occurring at the anode. The primary descriptor for efficient hydrogen production is thermoneutral Gibbs free energy of hydrogen atom adsorption, ideally close to 0 eV. This facilitates optimal adsorption and desorption of hydrogen gas.^[^ ^82^ ^]^ The HER mechanism can be categorized based on whether the hydrogen is supplied directly from the electrolyte or generated through the adsorption and recombination of intermediates. In acidic conditions, HER proceeds through either the Volmer–Heyrovsky mechanism $\left(H^{*}+H^{+}+e^{-}\to H_{2}\left(g\right)\right)$ or the Volmer–Tafel mechanism $\left(H^{*}+H^{*}+e^{-}\to H_{2}\left(g\right)\right)$. In alkaline conditions, the Volmer step provides protons via water dissociation, followed by either the Volmer–Heyrovsky process $\left(H^{*}+H_{2}O+e^{-}\to H_{2}(g)+{\text{OH}}^{-}\right)$ or the Volmer–Tafel process $\left(H^{*}+H^{*}+e^{-}\to H_{2}(g)+{\text{OH}}^{-}\right)$. For calculating proton and electron pairs in DFT simulations, the computational hydrogen electrode model utilizes half the energy of hydrogen gas.^[^ ^83^ ^]^ Importantly, acidic electrolytes offer an abundant source of protons, whereas alkaline electrolytes require an additional activation barrier for effective water dissociation and H_2_ generation.^[^ ^84^ ^]^

**Figure 5:**
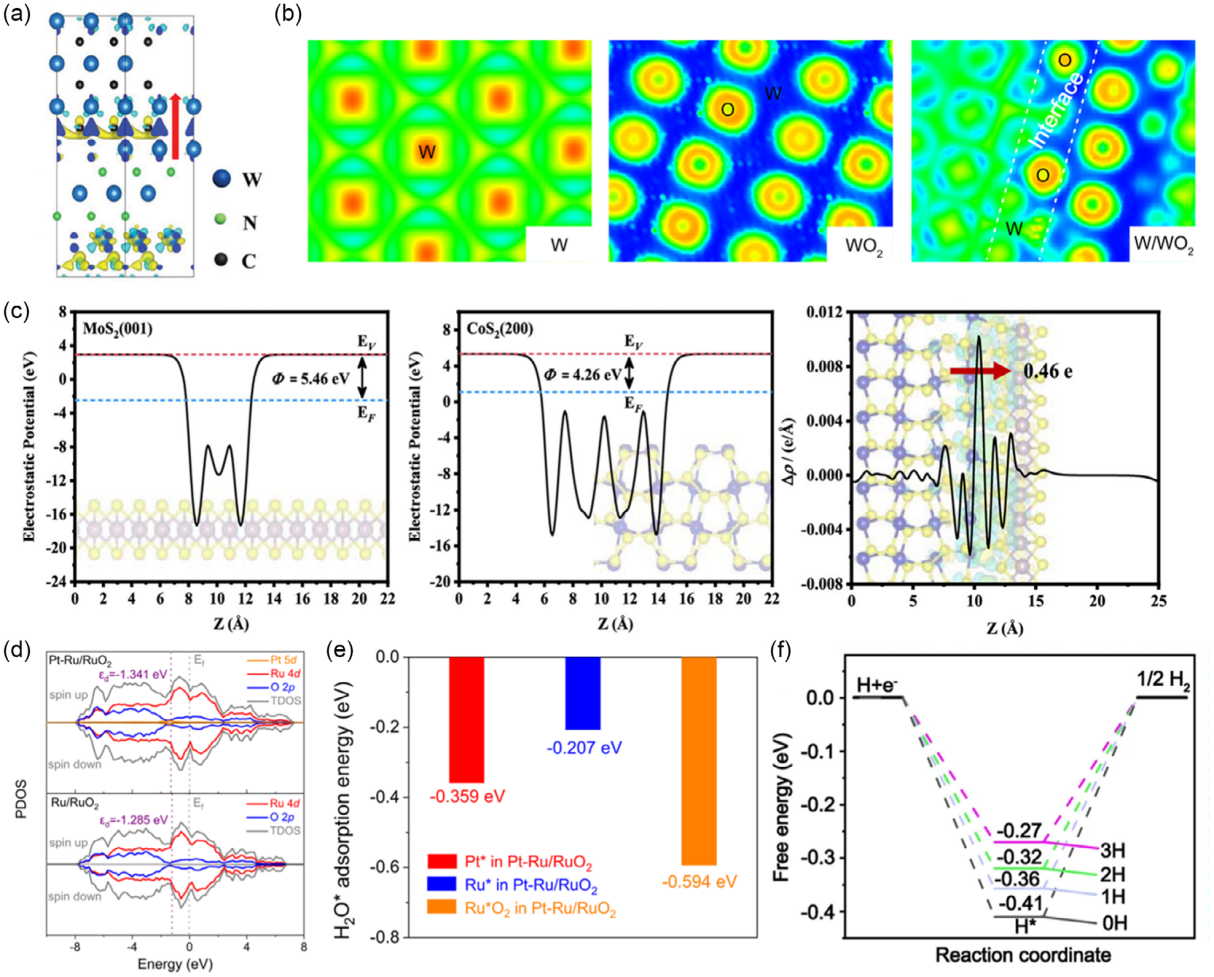
Electronic structure and activity analysis on HER heterostructured catalyst. a) CDD of WC/W_2_N. Reproduced with permission.^[^ ^55^ ^]^ Copyright 2019, WILEY‐VCH GmbH. b) ELF of W, WO_2_, and W/WO_2_. Reproduced under the terms of the Creative Commons CC BY license.^[^ ^39^ ^]^ c) The electrostatic potentials and work functions of MoS_2_, CoS_2_, and CoS_2_‐MoS_2_. Reproduced with permission.^[^ ^86^ ^]^ Copyright 2024, American Chemical Society. d) PDOS of Pt—Ru/RuO_2_, and Ru/RuO_2_. e) Gibbs free energy of H_2_O adsorption on Pt, Ru, and Ru of RuO_2_ sites in Pt–Ru/RuO_2_.^[^ ^40^ ^]^ Reproduced under the terms of the Creative Commons CC BY license. f) The differential hydrogen adsorption energies on W/WO_2_. Reproduced under the terms of the Creative Commons CC BY license.^[^ ^39^ ^]^

Substantial charge redistributions between components and electron transfers to active sites have significantly enhanced HER activity in various heterostructures. For example, Oh et al. developed a heterostructure of La_0.5_Sr_0.5_CoO_3−*δ*_ (LSC) combined with Ketjenblack carbon, which assisted the phase transition of MoSe_2_ from the low‐conductive 2H phase to the metallic 1T phase.^[^ ^23^ ^]^ This transition was further accelerated by incorporating potassium ions (K‐MoSe_2_), promoting greater charge accumulation on MoSe_2_.This accumulation is attributed to the difference in electronegativity between K and Se, which drives charge transfer from K to Se. Through atomic‐scale charge analysis, it was revealed that CoO_2_ within LSC predominantly transferred electrons to MoSe_2_, emphasizing the importance of CoO_2_ in electron transfer. This method allows for precise identification of materials contributing to electron transfer, providing crucial insights into optimizing catalytic performance. In addition, the electron accumulation in MoSe_2_ reduced the Gibbs free energy of hydrogen adsorption in LSC/K‐MoSe_2_ (0.25 eV), which indicated superior HER activity compared to LSC (2.01 eV), 2H‐MoSe_2_ (1.44 eV), and K‐MoSe_2_ (0.69 eV). Additionally, the electron‐donating Co in the heterostructure became electrophilic, raising the *d*‐band center and stabilizing OER intermediates, enabling LSC/K‐MoSe_2_ to function as a bifunctional electrocatalyst.^[^ ^85^ ^]^ Diao et al. synthesized a W_2_N/WC heterostructure by trapping CN_*x*_ on WO_*x*_ nanorod catalysts, followed by nitridation and carbonization. The superior activity of the WC/W_2_N was attributed to charge transfer from W_2_N to WC, as confirmed by CDD analysis, which showed electron accumulation near C atoms (**Figure** **5**a). This CDD result supported the potential of W_2_N/WC to function as a trifunctional catalyst for HER, OER, and ORR.^[^ ^55^ ^]^ Kumar et al. advanced alkaline HER catalysis by coupling the electronic structure of NiP_2_‐FeP_2_ with Cu. The introduction of Cu increased the charge density of P in NiP_2_–FeP_2_, improving the hydrogen adsorption Gibbs energy to −0.03 eV and reducing the water dissociation barrier to 0.16 eV. DFT calculations verified that Cu support accelerated HER activity via charge redistribution at the P sites.^[^ ^42^ ^]^ Finally, a study utilizing metals and metal oxides to enhance HER activity in alkaline environments effectively explained interfacial charge redistribution through ELF analysis. Chen et al. employed tungsten in oxidation states ranging from 0 to +6, highlighting its high electron utilization efficiency. In the W/WO_2_ heterostructured catalyst, WO_2_ functions as a Lewis acid, while H_*x*_WO_*y*_ and W serve as Brønsted acids. To address WO_*x*_ dissolution in alkaline environments, metallic W was introduced to form a dynamic proton‐concerted surface, enhancing HER kinetics. WO_2_ adsorbs and dissociates water, generating H_*x*_WO_*y*_, which can store protons. Zero‐valent W accelerated deprotonation of the H_*x*_WO_*y*_, facilitating hydrogen desorption. Although WO_2_ exhibited localized electrons and W showed delocalized electrons, ELF analysis revealed significant electron redistribution at the interface of W/WO_2_ heterostructure (Figure 5b). With its unique interfacial property, the W/WO_2_ heterostructure demonstrated a more thermoneutral hydrogen adsorption energy of −0.41 eV, compared to −1.24 eV for WO_2_ and −0.51 eV for W. Additionally, the study found that W/WO_2_ had similar water adsorption energy to WO_2_ but featured a lower water dissociation barrier, enhancing the water dissociation capability of WO_2_.^[^ ^39^ ^]^ Hence, charge redistribution analysis through CDD and ELF is an effective approach for improving HER activity, as it helps elucidate the origins of the optimal binding strengths in heterostructures.

DOS analysis, combined with charge density analysis, effectively evaluates HER activity. The Gibbs free energy of hydrogen adsorption needs to be close to 0 eV, requiring *d*‐band center optimization. Zhu et al. proposed a CoS_2_/MoS_2_@PPy catalyst for water splitting reaction, integrating MoS_2_ with CoS_2_ to promote water dissociation, with polypyrrole (PPy) enhancing conductivity. Electron transfer from CoS_2_ to MoS_2_ along with charge redistribution provided optimal adsorption energies for both HER and OER. The higher work function of MoS_2_ (5.46 eV) compared to CoS_2_ (4.26 eV) exhibited charge density accumulation in MoS_2_ (Figure 5c). DOS analysis also increased near the Fermi level in CoS_2_/MoS_2_, improving electrical conductivity.^[^ ^86^ ^]^ TM carbides, such as WC and Mo_2_C, are extensively used as HER catalysts due to their high conductivity, but their strong hydrogen binding strength hinders desorption, necessitating strategies to surpass performance of platinum.^[^ ^87^ ^]^ Liu et al. showed that the WC‐Mo_2_C heterostructure overcame this challenge through significant charge transfer at the interface. The similar physical and chemical properties of molybdenum and tungsten allowed the formation of a well‐matched W—Mo—C heterojunction. The heterostructure demonstrated favorable HER activity with an optimal Gibbs free energy of hydrogen adsorption (−0.04 eV), superior to WC (−1.02 eV) and Mo_2_C (−0.74 eV). PDOS analysis indicated that the lower *d*‐band center in WC‐Mo_2_C facilitated hydrogen desorption, with charge transfer from WC to Mo_2_C.^[^ ^88^ ^]^ Zhu et al. aimed to augment water splitting performance in alkaline electrolytes while complementing the moderate hydrogen production of Pt in HER. They developed a Pt single atom on Ru/RuO_2_ heterostructure, where RuO_2_ accelerated water dissociation, and the Pt single atom and metallic Ru promoted H recombination, resulting in an efficient catalyst. Electron transfer from Ru to RuO_2_ was observed, and the Pt—Ru/RuO_2_ had a lower *d*‐band center (−1.341 eV) compared to Ru/RuO_2_ (−1.285 eV), which weakened the hydrogen adsorption. Gibbs free energy comparison for hydrogen adsorptions at Pt, Ru, and RuO_2_ sites discovered that the Pt site had the most thermoneutral energy, accelerating H recombination and desorption (Figure 5d). In addition, water adsorption energies at Pt, Ru, and RuO_2_ sites further verified that RuO_2_ had the most stable energy, suggesting it as the active site for water dissociation (Figure 5e).^[^ ^40^ ^]^ Similarly, Xie et al. proposed the Ru/WO_3_ catalyst, where the WO_3_ behaved as Lewis acids for water adsorption and dissociation, with the generated hydrogen migrating to Ru, thereby improving hydrogen generation. They further explained that the formation of oxygen vacancies in WO_3_ enhances electron redistribution, boosting the activity of the heterostructured catalyst. Integrating metals with metal oxides in heterostructured electrocatalysts provides a synergistic approach to optimizing both hydrogen generation and water dissociation processes. They compared five models: Ru, WO_3_, WO_3_ with oxygen vacancy (WO_3_–V_O_), Ru/WO_3_, and Ru/WO_3_–V_O_. CDD analysis showed that Ru/WO_3_–V_O_ had the highest electron redistribution, facilitating H_2_ adsorption and desorption. PDOS analysis revealed higher electron density near the Fermi level for Ru/WO_3_–V_O_, indicating oxygen vacancies could enhance electron transfer. The higher *d*‐band center for Ru/WO_3_‐V_O_ accelerated HER kinetics, reducing energy barriers for water dissociation and H* desorption.^[^ ^89^ ^]^ These examples demonstrate that modulating electron density at the Fermi level and optimizing the *d*‐band center are essential for improving HER activity and achieving thermoneutral hydrogen adsorption energy in heterostructures. In conclusion, these studies offer insights into how charge and DOS analyses effectively explain HER activity by achieving optimal hydrogen adsorption energy and facilitating water dissociation.

HER catalytic activity can be assessed through more strategic and innovative methodologies. Considering the adsorption of multiple hydrogen atoms can be one of the most effective approaches, as this occurs in real environments when the Gibbs free energy for hydrogen atom adsorption is negative. This hydrogen coverage effect helps explain how increased hydrogen adsorption affects adsorption trends. While single‐hydrogen adsorption often represents HER activity as a key descriptor, multiple hydrogen atom adsorptions offer a more comprehensive perspective. In the W/WO_2_ system, sequential hydrogen adsorption was performed to simulate the effect of H_*x*_WO_*y*_, with the Gibbs free energy shifting from −0.41 eV with one hydrogen to −0.27 eV with three hydrogens. As the adsorption energies became increasingly thermoneutral, this highlighted the role of WO_2_ in moderating hydrogen adsorptions (Figure 5f).^[^ ^39^ ^]^ Perilla et al. evaluated differential hydrogen binding energy on hBN/Cu(111) using the equation of 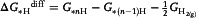, which calculates stepwise Gibbs free energy differences. In a boron vacancy model, the binding energy for a hydrogen atom was −1.53 eV, but as additional hydrogen atoms were adsorbed, the differential binding energy shifted to −0.49 eV (two hydrogens) and +0.02 eV (three hydrogens), indicating a more moderate state. Likewise, the BN vacancy model showed a shift from −1.13 eV for single hydrogen to −0.07 eV for two hydrogens, further illustrating how differential hydrogen binding energies with increasing hydrogen coverage could explain HER activity. For models with metals trapped at vacancy sites of hBN/Cu(111), the boron monovacancy model with a Ni atom on Cu(111) showed adequate hydrogen binding energy but exhibited a high energy barrier for hydrogen recombination, highlighting the need for kinetics analysis. As a result, they proposed the hBN with BN diatomic vacancy model with Fe on Cu(111), which demonstrated appropriate hydrogen binding energy and a lower Tafel step barrier, making it a promising catalyst under high hydrogen coverage conditions.^[22a]^ In conclusion, diverse strategies for HER activity analysis provide multiple perspectives for studying HER electrocatalysts.

### 5.3 Oxygen Reduction Reaction (ORR)

ORR is a critical process for advancing fuel cell technology during discharge. It offers an ecofriendly energy solution by producing water as a product. However, the sluggish kinetics of the four‐electron pathway present a significant challenge for ORR performance. In acidic conditions, ORR follows $O_{2}+4H^{+}+4e^{-}\to 2H_{2}O$, while in alkaline conditions, it proceeds via $O_{2}+2H_{2}O+4e^{-}\to 4{\text{OH}}^{-}$. Alternatively, if protons prefer to bond with oxygen adsorbed on surface, ORR may follow a two‐electron pathway, leading to the formation of hydrogen peroxide as a competing reaction. To enhance ORR performance, strategies are required to modulate the Gibbs free energies of *OOH, *O, and OH* intermediates to reduce the PDS.^[^ ^83^ ^]^ Consequently, extensive research has focused on utilizing heterostructure to modify the electronic structure and improve catalytic activity for ORR. However, if the adsorption strength of intermediates is too strong, the energy required for desorption may increase, reducing overall catalytic activity. For example, hBN/Ni(111) and hBN/Co(0001) exhibited lower OH adsorption energies than the overall Gibbs free energy of ORR (−4.92 eV), resulting in decreased ORR activity due to excessive adsorption. In contrast, hBN/Cu(111) showed that all Gibbs free energy steps became downhill at 0.89 V, with a theoretical overpotential of 0.34 V, comparable to Pt.^[22b]^ Therefore, achieving an appropriate adsorption energy is essential for high ORR performance. While precious metals like Pt verify their superior ORR activity, ongoing research explores alternative material systems to reduce reliance on them.^[^ ^90^ ^]^

**Figure 6:**
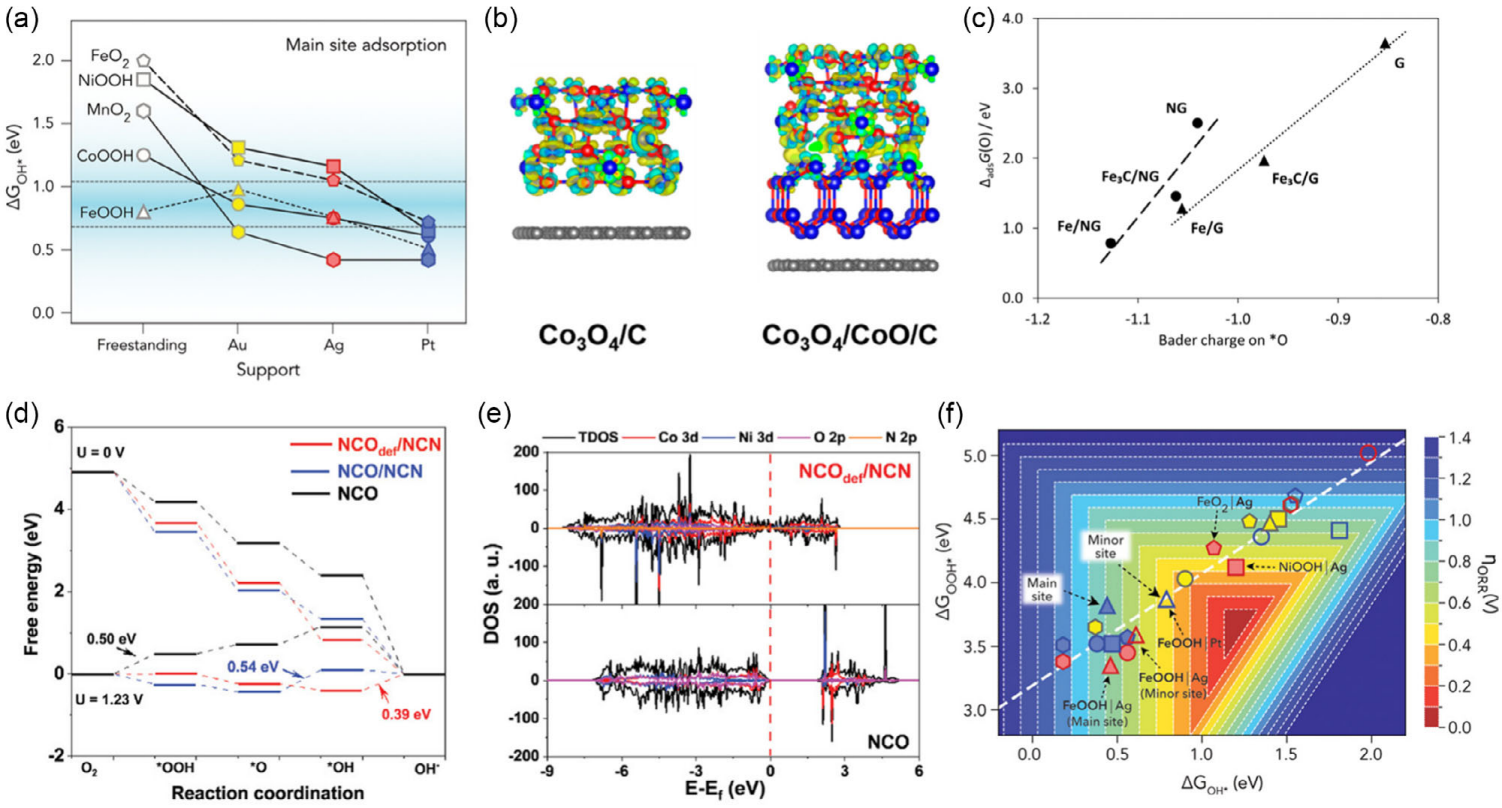
Electronic structure and activity analysis on ORR heterostructured catalyst. a) Gibbs free energy profile of OH adsorption on oxyhydroxide nanoribbons supported by noble metals. Reproduced with permission.^[^ ^34^ ^]^ Copyright 2019, American Chemical Society. b) CDDs in Co_3_O_4_/G and Co_3_O_4_/CoO/C. Reproduced with permission.^[^ ^27^ ^]^ Copyright 2023, American Chemical Society. c) The correlation between Bader charge of adsorbed oxygen and Gibbs free energy of O adsorption. Reproduced with permission.^[^ ^65^ ^]^ Copyright 2018, American Chemical Society. d) Gibbs free energy of the ORR on NCO_def_/NCN, NCO/NCN, and NCO. e) PDOS of NCO_def_/NDN and NCO. Reproduced with permission.^[^ ^92^ ^]^ Copyright 2023, WILEY‐VCH GmbH. f) A 3D volcano plot of oxyhydroxide nanoribbons with noble metals in terms of main site (filled symbol) and minor sites (empty symbol). Reproduced with permission.^[^ ^34^ ^]^ Copyright 2019, American Chemical Society.

Charge relocation at interface of ORR catalysts is a key factor in enhancing catalytic activity. In some cases, catalytic performance improves when charge accumulates at the active site, stabilizing adsorption energies and reaction intermediates. For example, the heterostructure of oxyhydroxide nanoribbons of Co, Fe, Mn, and Ni on noble metals (Au, Ag, and Pt) indicated that the oxophilicity of the support materials stabilizes the adsorption energy of intermediates. Utilizing OH adsorption free energy as an important descriptor for ORR, they identified that the adsorption energy was more stable with metal supports compared to individual oxyhydroxides and oxides (**Figure** **6**a). Bader charge analysis revealed that charge transfers from metal supports to oxides can contribute to the more stable adsorption. Consequently, NiOOH/Ag, FeO_2_/Ag, and CoOOH/Pt were proposed as promising catalysts for alkaline ORR, considering both activity and stability.^[^ ^34^ ^]^ Li et al. suggested a bifunctional electrocatalyst composed of CoO_*x*_ embedded in lamellar carbon nanofibers for both OER and ORR. They utilized covalent organic frameworks (COF) as carbon support and compared Co_3_O_4_/C and Co_3_O_4_/CoO/C catalysts to analyze mixed oxidation states of cobalt. CDD analysis showed electron accumulation on cobalt in Co_3_O_4_, which served as the active site, and substantial charge redistribution was observed (Figure 6b). This charge accumulation on Co_3_O_4_ was suggested to reduce the energy required for the PDS by optimizing OOH adsorption energy. It indicates that the optimized and combined oxidation state of CoO_*x*_ in COF‐based catalysts could contribute to excellent ORR performance.^[^ ^27^ ^]^ In addition, carbon materials offer advantages due to their low cost, high activity, and stability, particularly when forming graphitic layers.^[^ ^91^ ^]^ *N*‐doped graphene (NG) is especially effective, making it widely usable as an active site in ORR. Reda et al. investigated the effect of NG in iron carbide encapsulated in a graphitic layer, finding that the Fe_3_C/NG system exhibited a lower theoretical overpotential compared to Fe and graphene‐based heterostructures. In Fe_3_C/NG and Fe_3_C/G systems, charge transfer from carbide to NG compensates for the double bond property of O by filling the *sp* state, leading to stronger binding energy. Bader charge analysis of adsorbed oxygen showed a significant correlation with its adsorption energy. As more electrons accumulated on the adsorbed oxygen, the binding strength increased (Figure 6c). However, this correlation still requires further validation. Although electron transfer to graphene from carbide was higher in Fe_3_C/G compared to Fe/G, the adsorption was more stable in Fe/G. This indicates that surface electron transfer alone cannot fully explain adsorption strength, and multiple descriptors are needed to uncover the underlying factors. Therefore, when analyzing charge density, multiperspective approaches are essential to consider both surface systems and adsorbates. By integrating these aspects, it becomes possible to identify the factors that contribute to improved catalytic performance.^[^ ^65^ ^]^

On the other hand, charge depletion at the active site can also lead to improved activity by weakening certain adsorption energies and optimizing the reaction pathway. For instance, plasma‐assisted NiCoO/NiCoN (P‐NCO/NCN) supported on carbon fiber and carbon cloth was investigated for its bifunctionality of ORR and OER. Liu et al. compared the electronic structures of NCO_def_/NCN, NCO/NCN, and NCN to reveal the high activity of P‐NCO/NCN‐carbon fiber@carbon cloth. Both NCO/NCN and NCO_def_/NCN exhibited more stable Gibbs free energy compared to NCO alone, suggesting that heterostructure formation stabilized adsorptions. CDD analysis indicated electron transfer from NCO_def_ to NCN, increasing the metal oxidation states of NCO_def_. Bader charge analysis proposed that Co near an oxygen vacancy in NCO_def_ had a lower electron density (+1.36 |e|) compared to Co in NCO/NCN (+1.30 |e| ). Interestingly, the NCO_def_/NCN, which had lost more electrons, demonstrated more stabilized OH adsorption but weakened O and OOH adsorptions compared to NCO/NCN (Figure 6d). This altered the PDS and emphasized the importance of analyzing the entire reaction mechanism. Therefore, various intermediates within the reaction pathway influence the charge redistribution, and the energy stabilization varies depending on the adsorbates.^[^ ^92^ ^]^ MXene, known for its excellent electrical conductivity, has also been utilized as an ORR catalyst, particularly in heterostructures with carbon‐based materials. Zhou et al. explored the heterostructures of *N*‐doped graphene (NG) with MXenes (Ti_2_C, V_2_C, Nb_2_C, Mo_2_C). These heterostructures were expected to form stable interactions with adsorbates due to asymmetrical electron density and surface distortion. NG was found to provide multiple charge states depending on the N doping site, with the C atom adjacent to N at the hollow site of the support metal identified as the most stable active site. C atoms with lower electron density were suggested to stabilize ORR intermediates. As a result, NG on V_2_C and Mo_2_C exhibited low overpotentials of 0.36 and 0.39 V, respectively, while Nb_2_C and Ti_2_C had higher overpotentials of 0.54 and 0.64 V. Despite this, all these heterostructures outperformed freestanding NG, which had an overpotential of 1.24 V, indicating developed ORR activity in the heterostructure. To further analyze ORR kinetics, nudged elastic band calculations were performed to determine the barriers for O_2_ dissociation and OOH formation on promising NG/V_2_C and NG/Mo_2_C. The analysis revealed that NG/V_2_C required a lower activation barrier compared to NG/Mo_2_C. In addition, they found that overly electron depletion on the C atom by F atom doping could reduce activity, highlighting the significance of maintaining appropriate charge levels on active sites. Analysis of M_3_C_2_ instead of M_2_C in the heterostructures also showed similar performance, providing insight that heterostructures of 2D metal carbide and NG can serve as excellent electrochemical ORR catalysts.^[^ ^52^ ^]^ In summary, we examined examples and interpretations of how charge transfer influence ORR activity and highlighted both the benefits of charge accumulation and depletion at active sites.

Based on the previous examples, it is evident that explaining catalytic activity requires a multifaceted approach. Analyzing the DOS in these systems provides a deeper understanding of catalytic performance. For instance, when CoOOH forms a heterostructure with metals, the binding energy of OH weakens in the order of Pt, Ag, and Au. This phenomenon was linked to the oxophilicity of the metals and the antibonding states filling, as observed through DOS analysis. The fillings of antibonding state reduce interactions with adsorbates, and *d*‐band centers near the Fermi level provide more stable active sites. Specifically, in Au, the most noble metal, the antibonding states were filled and positioned below the Fermi level, resulting in weaker interactions with adsorbates compared to Ag and Pt.^[^ ^34^ ^]^ Additionally, NCO_def_/NCN displayed a disappearance of the bandgap in its DOS analysis compared to NCO. This indicated that while NCO exhibited semiconducting properties, forming a heterostructure with NCN alters its electronic structure. The higher electron density at the Fermi level also promoted the electrical conductivity of heterostructure (Figure 6e).^[^ ^92^ ^]^ Similarly, the MXene/NG heterostructure exhibited dominant electron density at the Fermi level, supporting a more conductive state. The *p*_*z*_‐band center of carbon in the graphitic layer also serves as a useful descriptor. As the *p*_*z*_‐band center approaches the Fermi level, its involvement in *π* bonding leads to higher‐energy states, which weakens OH binding strength.^[^ ^52^ ^]^ This relationship was also observed in NG combined with Fe_3_C and metal‐substituted Fe_3_C, where the *p*_*z*_‐band center of carbon atoms in NG correlated with the adsorption energy of intermediates.^[^ ^64^ ^]^ Moreover, the shift in the *p*_*z*_‐band center is related to the work function. As the work function increased, the bands of NG shifted to lower energy levels relative to the Fermi level. Thus, a larger work function correlates with a lower *p*_*z*_‐band center and stronger binding energy.^[^ ^52^ ^]^ Consequently, maintaining a broad perspective that includes material‐specific descriptors is important for understanding and improving catalytic activity across different systems.

In ORR, the binding energies of intermediates are interdependently connected, influencing the extent of performance improvements. The OOH and OH binding energies on metals and metal oxides represent a scaling relationship of ΔG(*OOH)=ΔG(*OH)+3.2 eV.^[^ ^89^ ^]^ As a result, a volcano plot can be constructed using the theoretical overpotentials and the free energies of oxygen intermediates as descriptors. Even at the summit of a volcano plot, a minimal theoretical overpotential is present. Therefore, efforts to break the scaling relationship and improve performance are ongoing. Various scaling relationships and volcano plots for different heterostructures in ORR have been reported. In the Fe_3_C/NG system, the relationship ΔG(*OOH)=ΔG(*OH)+3.4 eV was observed.^[^ ^65^ ^]^ In the NG/substituted metal in Fe_3_C system, the relationships ΔG(*OOH)=ΔG(*OH)+3.5 eV and ΔG(*O)=2ΔG(*OH)−0.47 eV were observed for catalysts that maintained structural integrity.^[^ ^64^ ^]^ In NG/MXene system, OH adsorption Gibbs free energy was used as a descriptor in a 2D volcano plot. On the left side, strong adsorption makes OH desorption the PDS, while on the right side, weak adsorption strength makes O_2_ dissociation to form OOH the PDS. This volcano plot helped identify the PDS for other NG/MXene heterostructures. By establishing volcano plots based on catalytic characteristics, high‐throughput screening can utilize a single‐adsorption energy to gain insights into the PDS for numerous candidate materials. Furthermore, the ORR activity of oxyhydroxide/metal systems followed a scaling relationship of ΔG(*OOH)=0.88ΔG(*OH)+3.19 eV at main sites. Based on the 3D volcano plot for ORR activity, CoOOH/Au, NiOOH/Ag, and FeO_2_/Ag were identified as the best candidates for the main sites, whereas CoOOH/Pt and FeOOH/Pt were superior for the minor sites only regarding activity. These catalysts were positioned near the volcano summit, demonstrating an optimal balance of Gibbs free energy for high catalytic performance (Figure 6f).^[^ ^34^ ^]^

### 5.4 Carbon Dioxide Reduction Reaction (CO_2_RR)

CO_2_RR offers a promising solution to environmental and energy challenges by converting CO_2_, a major greenhouse gas, into valuable hydrocarbons and oxygenates through electrochemical processes. Utilizing CO_2_ and protons, CO_2_RR produces products such as carbon monoxide (CO), methane (CH_4_), and ethylene (C_2_H_4_), which can be utilized as valuable resources in industry and as clean energy carriers.^[^ ^93^ ^]^ The typical two‐electron CO_2_RR is divided based on protonation sites: either forming CO (${\text{CO}}_{2}+2H^{+}+2e^{-}\to \text{CO}+H_{2}O$) or formic acid (${\text{CO}}_{2}+2H^{+}+2e^{-}\to \text{HOCHO}+H_{2}O$). However, CO_2_RR faces critical challenges, including high overpotentials and selectivity issues due to competing HER.^[^ ^94^ ^]^ The complex nature of CO_2_ reduction, involving multiple electron transfers, complicates achieving high Faradaic efficiencies and selectivity for desired products. Additionally, electrocatalysts often suffer from poor stability, which limits their potential for large‐scale CO_2_ conversion. Noble metal catalysts like silver and gold have proven effective in promoting CO_2_RR, but their high cost and limited availability present economic and scalability challenges.^[^ ^95^ ^]^ In response, non‐noble metal alternatives, such as carbon‐ and copper‐based catalysts, are being explored for their cost‐effectiveness and stability.^[^ ^96^ ^]^ Recent studies focus on the development of advanced catalysts, with particular attention to heterostructured catalysts that integrate multiple materials. These catalysts are designed to optimize active site distributions and reaction pathways and promote CO_2_RR performance.

Accurately defining the scope and role of CDD, especially their involvement at active sites, is crucial for understanding catalytic performance. Yin et al. utilized M–N_4_ catalysts, known for their high electrical conductivity and tunable bandgaps, as alternatives to noble metals.^[^ ^97^ ^]^ However, Fe—N sites exhibit strong binding affinity for CO and H, making CO desorption challenging. To address this, a FeN/Fe_3_N nanoparticle heterostructure was proposed, with strategically distributed Fe–N_4_ and Fe–N_2_ sites. CDD analysis revealed electron transfer from Fe_3_N to FeN, favoring CO_2_ adsorption and its reduction to COOH on FeN. This was attributed to the difference in work function between FeN (6.56 eV) and Fe_3_N (5.41 eV), resulting in a built‐in electric field from Fe_3_N to FeN. The study highlighted the importance of charge density redistribution at the surface. Furthermore, comparing different facets, FeN(100)/Fe_3_N(001) exhibited excellent activity and observable electron transfer to the active sites, whereas FeN(110)/Fe_3_N(001) showed no redistribution of electrons across the interface. This emphasizes the necessity of accurately modeling heterostructures to reflect interfacial characteristics.^[97a]^ Wang et al. demonstrated the synergistic effects of C_3_N_4_/G and cobalt phthalocyanine (CoPc). C_3_N_4_/G promoted CO_2_ adsorption and water dissociation, facilitating proton formation, while CoPc reduced the energy barrier for converting CO_2_ to COOH. The combination of C_3_N_4_/G with CoPc accelerated the thermodynamic conversion of CO_2_. CDD analysis revealed that electrons accumulated on C_3_N_4_/G, while CoPc tended to lose electrons. The C_3_N_4_/G support stably adsorbed CO_2_ and the CoPc/C_3_N_4_/G facilitated spillover to Co site.^[^ ^98^ ^]^ In the case of formic acid formation, *p*‐block metal catalysts, including indium and bismuth, have been explored for their high selectivity. Zhao et al. proposed an In_2_O_3_/InN heterostructure to enhance the activity and stability of indium‐based catalysts. ELF analysis confirmed that the N—O bond at the interface facilitated charge transfer between the materials, forming covalent bonds. CDD analysis showed electron transfer from In_2_O_3_ to InN, leading to electron enrichment in InN. Furthermore, the In_2_O_3_/InN heterostructure favored the formation of OCHO over COOH, explaining the selectivity of indium‐based catalysts for formic acid. Comparing the pathways to CO and formic acid (HOCHO), it was found that OCHO* formation from CO_2_ is exergonic, while COOH* formation is endergonic. For the side reaction HER, the heterostructure showed weaker water adsorption energy and a higher Gibbs free energy for hydrogen generation compared to In_2_O_3_, making In_2_O_3_/InN an effective catalyst for CO_2_RR to formic acid.^[^ ^99^ ^]^ Thus, understanding the role of each material in the heterostructure and analyzing both CO_2_RR selectivity and HER activity as a side reaction further support these catalysts’ performance.

**Figure 7:**
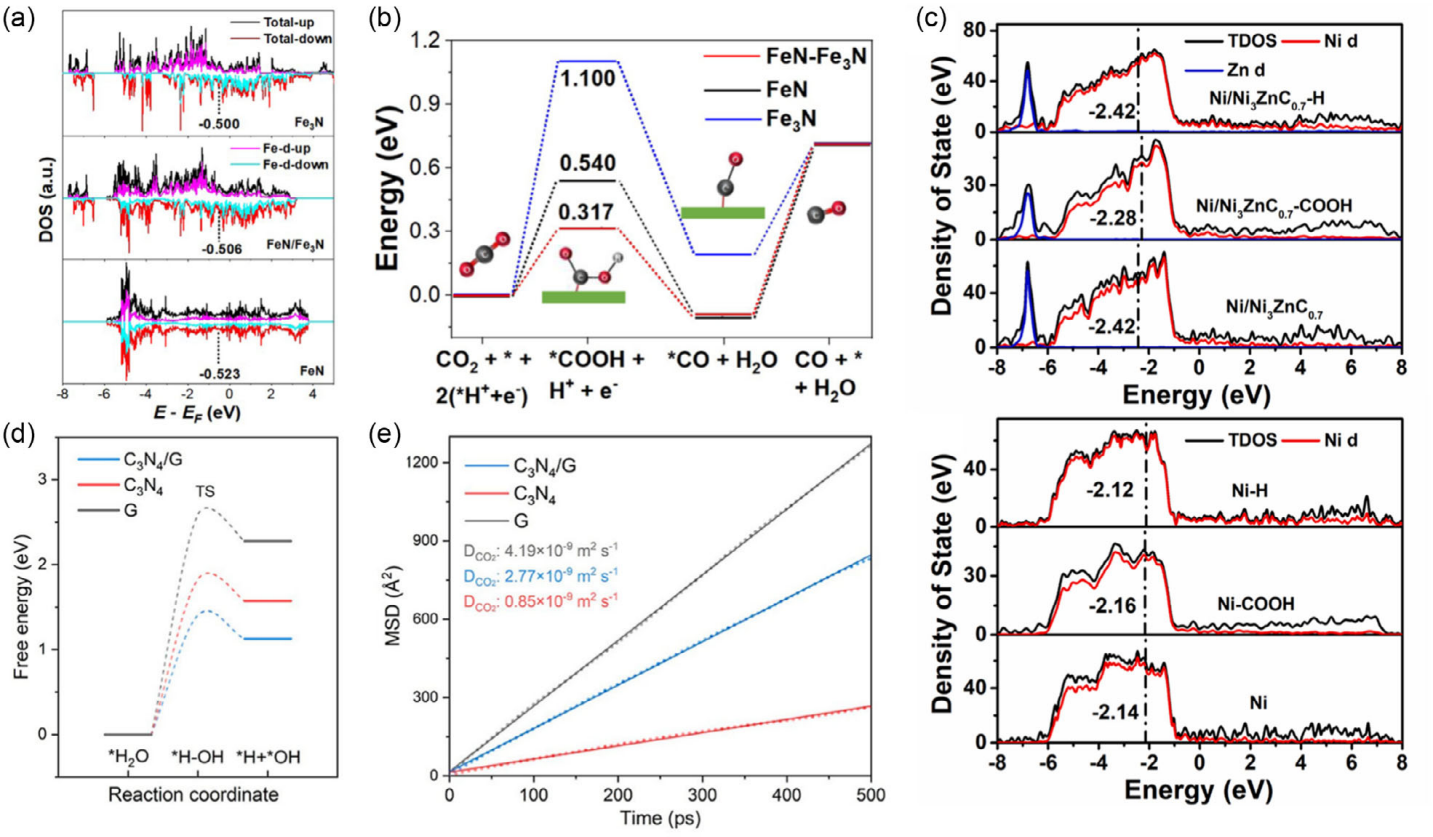
Electronic structure and activity analysis on CO_2_RR heterostructured catalyst. a) PDOS. b) Gibbs free energy of the CO formation on Fe_3_N site, FeN site, and FeN/Fe_3_N surface in the heterostructure. Reproduced under the terms of the Creative Commons CC BY license.^[97a]^ c) PDOS of Ni/Ni_3_Zn_0.7_ and Ni. Reproduced with permission.^[^ ^101^ ^]^ Copyright 2021, Elsevier. d) Gibbs free energy of H_2_O dissociation. e) Mean squared displacement and diffusion coefficients of C_3_N_4_/G, C_3_N_4_, and G. Reproduced with permission.^[^ ^98^ ^]^ Copyright 2024, American Chemical Society.

In CO_2_RR to CO, the formation of the COOH intermediate is usually considered as the PDS, and *d*‐band center analysis helps uncover factors that improve catalytic efficiency. Detailed electronic structure analysis of the FeN(100)/Fe_3_N(001) heterostructure demonstrated significant charge distribution, with an overall *d*‐band center of −0.506 eV. The individual *d*‐band centers were −0.523 eV for FeN(100) and −0.500 eV for Fe_3_N(001), indicating a synergetic upshift of *d*‐band centers closer to the Fermi level (**Figure** **7**a). Additionally, the heterostructure achieved a higher electron density at the Fermi level compared to the individual structures, facilitating stable interaction with adsorbates. The heterostructure showed the COOH formation energies of FeN site (0.540 eV), Fe_3_N site (1.10 eV), and interface (0.317 eV), suggesting high activity at the interface (Figure 7b).^[97a]^ The heterostructure provides a range of active sites, allowing for the site‐specific activity comparison and atomic‐scale analysis of catalytic activity. Ni—N—C‐based catalysts are also being investigated for CO_2_RR due to their superior activity and stability.[^97^, ^100^] However, these catalysts struggle to maintain long‐term activity due to competition with HER. To address this, various Ni‐based catalysts are being explored to reduce HER selectivity. Wei et al. synthesized a Ni/Ni_3_ZnC_0.7_ catalyst, which achieved superior CO production compared to Ni—N—C. The Ni(111)/Ni_3_ZnC_0.7_(100) heterostructure required 1.35 eV for COOH formation in the CO_2_RR pathway, showing higher activity than Ni. Moreover, it had an energy barrier of 0.27 eV for the competing HER, less favorable than the 0.13 eV for Ni. The difference in catalytic performance was driven by the *d*‐band center shift upon COOH adsorption. The Ni/Ni_3_ZnC_0.7_ exhibited an upshift in the *d*‐band center from −2.42 to −2.28 eV upon COOH adsorption, indicating a strong interaction with the adsorbate. In contrast, the Ni catalyst showed a downshift in the *d*‐band center (Figure 7c).^[^ ^101^ ^]^ Similarly, in the CoPc/C_3_N_4_/G catalyst, *d*‐band center analysis of Co upon COOH adsorption revealed that CoPc/C_3_N_4_/G was significantly closer to the Fermi level compared to CoPc/C_3_N_4_ and CoPc/G. This upshift supported strong interaction with the adsorbate, reducing the energy required for COOH adsorption. Additionally, water dissociation energy barriers were calculated, confirming that CoPc/C_3_N_4_/G had the lowest barrier for proton supply (Figure 7d). To verify the dynamics of CO_2_, MD simulations found that the diffusion coefficient for CO_2_ migration was highest in G, followed by C_3_N_4_/G, and C_3_N_4_ (Figure 7e). CO_2_ radial distribution function analysis indicated that CO_2_ adsorbed first on C_3_N_4_, followed by C_3_N_4_/G, and finally G, which matched isotherm and temperature‐programmed desorption results. This suggests that C_3_N_4_ facilitated initial CO_2_ adsorption. AIMD simulations confirmed the structural stability at 298.15 K. Thus, a thorough analysis of catalytic properties, both of the support alone and in combination with CoPc, was performed using various computational methods, including DFT, MD, and AIMD.^[^ ^98^ ^]^ A notable aspect is the comparison of *d*‐band center shifts in adsorbed states rather than pristine surface systems. Additionally, the rationales behind selectivity were reinforced by analyzing the competing HER in CO_2_RR.

Interestingly, cluster‐based models are also widely used in CO_2_RR to analyze the heterostructures and heterojunctions of electrocatalysts. For instance, the Cu/CeO_2−*x*_ catalyst, modeled using a Ce_6_O_13_ cluster, broke the scaling relationship between CHO and CO, thereby lowering the barrier for bidentate adsorption.^[^ ^102^ ^]^ Additionally, the modeling of amorphous CeO_2_ and Cu heterostructure was done by embedding 24 atoms of Cu(111) on CeO_2_(110) for multicarbon alcohols.^[^ ^103^ ^]^ The heterostructure formed by Cu(111) and a Sn_6_O_12_ cluster was proposed to shift the preferred intermediate formation from OCHO to COOH.^[^ ^104^ ^]^ Li et al. explored heterostructures formed by Cu and various metal oxides, such as ZrO_2_, HfO_2_, Zr_2_O_3_, SiO_2_, and Ga_2_O_3_. They modeled ZrO_2_/Cu as a proof catalyst by placing a Zr_4_O_8_ cluster on Cu(111), which stabilized CO_2_ and CO adsorption and promoted the C^2+^ pathway with a preference for OCCO intermediate over COH.^[^ ^105^ ^]^ Continued progress in catalyst design and fundamental studies on heterostructures will pave the way for fully understanding the CO_2_RR mechanism and producing valuable products.

### 5.5 Nitrogen Reduction Reaction (NRR)

The electrochemical NRR is highly promising for sustainable ammonia production, crucial for agriculture and various industries.^[^ ^106^ ^]^ The NRR converts nitrogen and protons into ammonia, typically represented by the reaction.

(16) $$N_{2}+6H^{+}+6e^{-}\to 2{\text{NH}}_{3}$$

Operating under ambient conditions, NRR offers a more energy‐efficient alternative to the traditional Haber–Bosch process, which requires high temperatures and pressures.^[^ ^107^ ^]^ However, challenges include high overpotential and low Faradaic efficiency due to the complex six‐electron transfer required to reduce N_2_, which has a strong triple bond (941 kJ mol^−1^).^[^ ^108^ ^]^ The NRR mechanism is intricate and not fully understood, with several proposed pathways. The NRR can follow associative or dissociative pathways, with specific mechanisms such as distal and enzymatic pathways, involving stepwise hydrogenation of N_2_ to NH_3_. The process often produces nitrogenous side products, including NO_*x*_ species, which complicate the reaction. In many studied catalysts, the formation of intermediates such as *NNH, *NH_2_, and *NH_3_ is crucial, with the PDS frequently involving the adsorption and activation of N_2_.^[^ ^108^ ^]^ This strong binding to active sites leads to high activation energies, resulting in slow kinetics and low catalytic activity. To overcome these challenges and improve the selectivity and activity of NRR catalysts, researchers have explored various materials.

**Figure 8:**
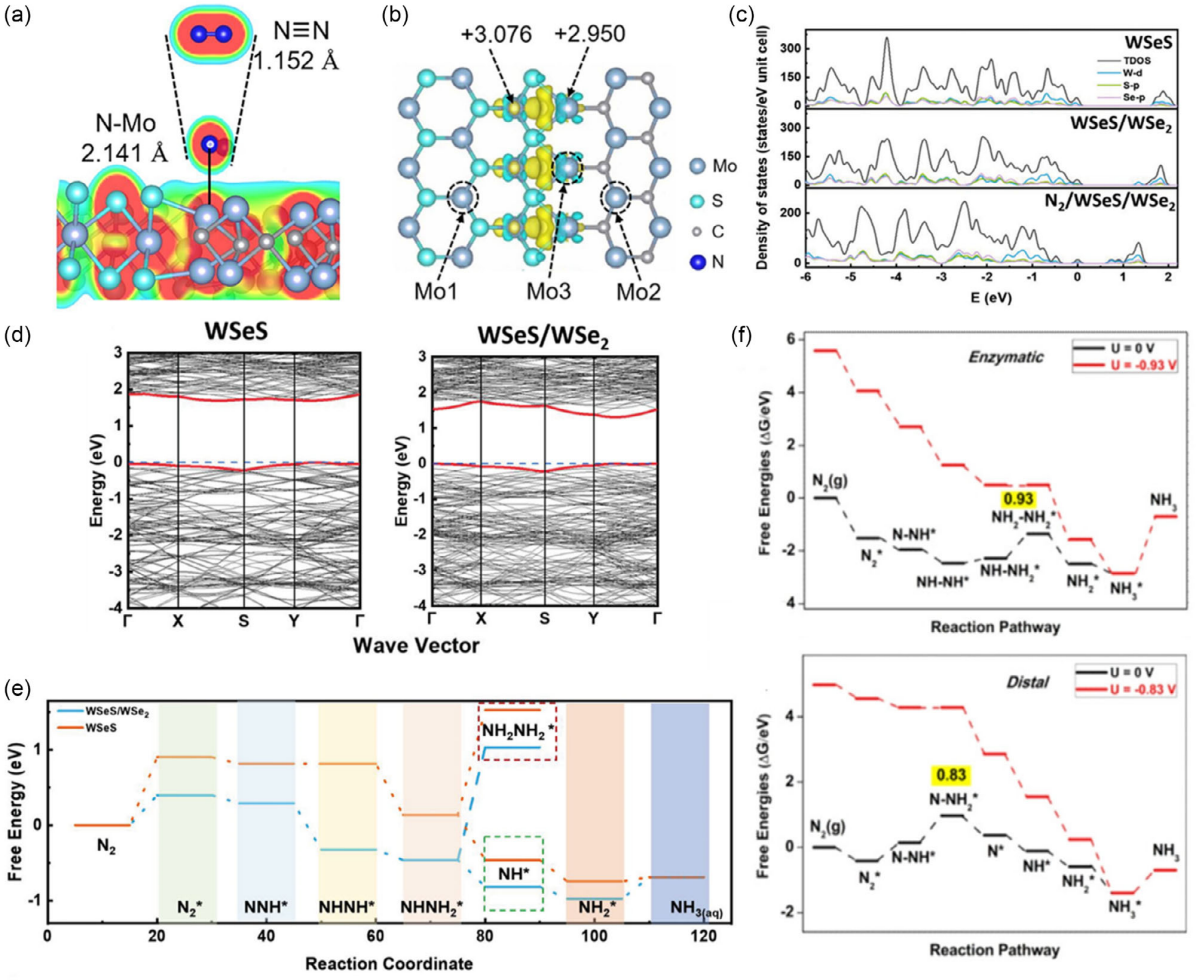
Electronic structure and activity analysis on NRR heterostructured catalyst. a) Contour charge map for N_2_ adsorption on the top Mo site on the interface of MoS_2_/Mo_2_C heterostructure and the corresponding N≡N bond lengths and Mo—N distances. b) CDD of MoS_2_/Mo_2_C heterostructure at the interface.Reproduced with permission.^[^ ^109^ ^]^ Copyright 2022, Elsevier. c) TDOS and PDOS of Janus WSeS, Janus WSeS/WSe_2_, and N_2_ adsorption on Janus WSeS/WSe_2_. d) Band structure of Janus WSeS and WSeS/WSe_2_ heterostructure nanowalls. With the introduction of the heterostructure, a new band appeared. e) The Gibbs free energy of the NRR on Janus WSeS and WSeS/WSe_2_ heterostructure nanowalls. Reproduced with permission.^[^ ^112^ ^]^ Copyright 2023, Wiley‐VCH GmbH. f) The Gibbs free energy diagrams for the NRR on Fe_3_C/Fe_3_O_4_ and O—Fe_3_C/Fe_3_O_4_ at the applied potential. The asterisk (*) represents a surface site for adsorption, and the maximum ΔG of the pathways is highlighted in yellow. Reproduced with permission.^[^ ^114^ ^]^ Copyright 2023, WILEY‐VCH GmbH.

While noble metals like Ru and Pt are effective, their high cost has prompted interest in non‐noble metal catalysts and heterostructured catalysts. These materials offer unique properties that improve catalytic activity through increased active sites and operative charge transfer. The electrocatalytic activity of NRR is driven by electron dynamics of two materials at the interface of heterostructured catalyst, where charge transfer can optimize efficiency. Ye et al. investigated charge transfer by calculating the CDD and using contour charge maps for N_2_ adsorption on the Mo site at the interface. The contour charge map represents accumulation of electrons with red region and depletion of electrons with blue region (**Figure** **8**a). Bader analysis of a MoS_2_/Mo_2_C catalyst, which exhibited a valence state ranging from Mo^2+^ to Mo^4+^, revealed that Mo^3+^ sites at the interface had a stable N_2_ adsorption energy of −0.75 eV in a side‐on configuration, facilitating the activated hydrogenation of *NH_2_ species. The CDD map clearly indicated significant electron migration from Mo_2_C to MoS_2_ across the interface. This interfacial electron transfer resulted in electron accumulation on MoS_2_ (yellow region) and electron depletion on Mo_2_C (cyan region). Bader analysis further quantified the electron transfer values, showing that the Mo_2_C lost 0.950 electrons while MoS_2_ gained 0.924 electrons (Figure 8b).^[^ ^109^ ^]^ Similarly, Tang et al. evaluated CDD in BN/V/G systems and highlighted the ‘acceptance‐donation’ mechanism of electrons, which is crucial for N_2_ adsorption and activation in TM‐based catalysts. In BN/TM/G systems, the B atom near the TM site primarily adopts *sp* ^*2*^ or partially *sp* ^*3*^ hybridization due to deformation. The TM atom donated electrons to the B atom, occupying partial *p*_*z*_ orbitals, while the remaining *p*_*z*_ orbitals accepted electrons from N_2_. This interaction strengthens bonding and weakens the N≡N triple bond through a ‘donation–backdonation’ mechanism, consistent with other boron‐based catalysts.^[^ ^110^ ^]^ Additionally, Tan et al. calculated partial charge density of the highest occupied orbitals below the Fermi surface and the lowest unoccupied molecular orbital above the Fermi surface of MoO_2_@MoO_3_ to explain newly formed electronic states in the PDOS. When N_2_ adsorbs on the surface, charge transfer occurs from N_2_ to Mo^4+^ through σ donation, shortening the N≡N bond and making it harder to dissociate for MoO_2_. In MoO_2_@MoO_3_ interface, charge transfer happened through *π* back‐donation, with Mo^4+^ and Mo^6+^ ions binding to single‐nitrogen atoms. Asymmetric *π* back‐donation at the interface enhanced Mo–N_2_ interactions, significantly boosting NRR activity. This strategic analysis is crucial for pinpointing electron transfer regions as main sites for ammonia adsorption, emphasizing the complex interaction of electronic properties in boosting catalytic efficiency.^[^ ^111^ ^]^

Moreover, PDOS analysis can provide essential insights into the catalytic mechanisms of heterostructured catalysts used in NRR. PDOS highlights how changes in band structure and the Fermi level can impact NRR activity of these catalysts. For example, MoO_2_@MoO_3_ represents newly formed band structures in the PDOS, where charge analysis of N_2_ adsorption onto Mo^4+^ and Mo^6+^ ions occurs through *π* back‐donation. This results in narrowing of the bandgap, contributing to improved catalytic performance.^[^ ^111^ ^]^ Similarly, Peng et al. conducted N_2_ adsorption calculations on Janus WSeS, WSeS/WSe_2_, along with band structure analysis. According to the PDOS calculations, when N_2_ adsorbs, a hybridization of W's *d*‐orbitals and N_2_'s *p*‐orbitals occurs near the Fermi level. This *p*–*d* orbital hybridization strengthened the binding between *W* and the coordinated nitrogen, leading to effective nitrogen adsorption on the WSeS/WSe_2_ heterostructure and resulting in efficient NRR (Figure 8c). Additionally, the bandgap was calculated to be 1.5 eV for WSeS/WSe_2_ and 1.8 eV for WSeS, with the decrease in bandgap indicating enhanced activity by promoting electron transfer through increased charge carriers (Figure 8d).^[^ ^112^ ^]^ PDOS analysis is essential for understanding electron transfer, revealing that new electronic states are created due to the heterostructure formed by different materials. The narrowing of the bandgap directly indicates increased electronic conductivity, which subsequently leads to enhanced activity. In summary, stable N_2_ adsorption energy is usually considered as a key factor for high‐performance NRR, as demonstrated through CDD and PDOS analyses. With many researchers focusing on N_2_ itself, detailed interpretations of N_2_ adsorption are supported by charge transfer between heterostructured materials, hybridization of orbitals, and distinctive DOS and band structures. Understanding these factors is essential for optimizing NRR catalytic activity and facilitating N_2_ activations.

NRR can be broadly categorized into associative ($N_{2}\to N_{2}H_{2}\to N_{2}H_{4}\to 2{\text{NH}}_{3}$), where N_2_ remains intact during the early stages of hydrogenation, and dissociative pathways ($N_{2}\to {\text{NH}}_{3}+N\to {\text{NH}}_{3}+\text{NH}\to {\text{NH}}_{3}+{\text{NH}}_{2}\to 2{\text{NH}}_{3}$), where N_2_ is split into individual nitrogen atoms first. Associative mechanism can be divided into distal ($N_{2}\to N_{2}H\to N_{2}H_{2}\to {\text{NH}}_{2}\text{NH}\to {\text{NH}}_{3}+\text{NH}\to 2{\text{NH}}_{3}$), alternating ($N_{2}\to N_{2}H\to N_{2}H_{2}\to N_{2}H_{3}\to {\text{NH}}_{3}$), and enzymatic pathways $\left(N_{2}\to N_{2}H\to N_{2}H_{2}\to {\text{NH}}_{3}+N\to {\text{NH}}_{3}+\text{NH}\to {\text{NH}}_{3}+{\text{NH}}_{2}\to 2{\text{NH}}_{3}\right)$.^[^ ^113^ ^]^ Researchers select the appropriate pathway for their heterostructured catalyst to calculate NRR intermediates, but note that resulting intermediates can vary depending on the choice of catalyst. These intermediates are then used to establish reaction pathways and identify the PDS, which is subsequently compared with those of individual catalysts. Ye et al. investigated NRR mechanism based on the associative pathway on MoS_2_/Mo_2_C heterostructure. During N_2_ fixation, the N_2_ molecule adsorbed onto different Mo sites, significantly affecting bond lengths and activation. The triple N bond length elongated at the Mo3 site, indicating more favorable activation compared to Mo1 and Mo2 sites. The Gibbs free energy of N_2_ adsorption was −0.75 eV at Mo3, much lower than on Mo1 and Mo2 sites. At the Mo1 site, the PDS was the hydrogenation of NHNH (*NHNH→*NHNH_2_), while at the Mo2 sites, the PDS was the hydrogenation of N_2_ (*N_2_ → *N_2_H), both of which were endergonic. In constrast, at the Mo3 site, these steps became exergonic, and the PDS shifted to the hydrogenation of NH_2_, with a significantly lower energy barrier, demonstrating its favorable NRR activity as the most active site.^[^ ^109^ ^]^ Furthermore, Peng et al. evaluated both associative and dissociative pathways for the Janus WSeS/WSe_2_ heterostructure, identifying the PDS as the formation of N with an energy barrier of 0.397 eV. The PDS energies for WS_2_ and WSe_2_ were 1.147 and 0.737 eV, respectively, indicating that the Janus WSeS/WSe_2_ heterostructure had a significantly lower energy barrier, enhancing its catalytic efficiency (Figure 8e).^[^ ^112^ ^]^ Yang et al. explored the distal and enzymatic pathways for the Fe_3_C/Fe_3_O_4_ heterostructure. Their DFT calculations revealed a preference for the distal pathway, which involved side‐on adsorption of N_2_ and sequential hydrogenation of each nitrogen atom. The PDS in the distal pathway was the formation of the N–NH_2_* intermediate, requiring a relative Gibbs free energy of 0.83 eV, making it more favorable than the enzymatic pathway, which showed a higher‐energy requirement of 0.93 eV at the NH_2_–NH_2_ formation step. The distal pathway barrier was much lower than those in traditional NRR catalysts, where the formation of N or hydrogenation steps typically presented higher energy barriers. The study found that the Fe_3_C/Fe_3_O_4_ heterostructure favored the distal mechanism due to its lower energy barriers and efficient hydrogenation process (Figure 8f).^[^ ^114^ ^]^ Tang et al. investigated the distal and alternating pathways for the BN/V/G heterostructure. For this system, the PDS was identified as the formation of the N–NH intermediate with a relative Gibbs free energy of 0.89 eV. This PDS was distinct from traditional NRR catalysts where the PDS involved the N formation or other hydrogenation steps with higher energy barriers. The BN/TM/G heterostructure benefited from the synergistic effects of combining TM atoms with hBN and graphene, enhancing catalytic activity.^[^ ^110^ ^]^ Calculating NRR intermediate adsorption configurations and understanding the associated energy differences are crucial for comprehending the mechanistic changes and catalytic enhancements provided by heterostructured catalysts. Researchers should select the appropriate reaction pathway based on the materials used for the catalyst. The combination of two different materials can alter the preferred reaction pathway and the PDS. This variation is significant and must be carefully considered to optimize catalyst performance and improve catalytic efficiency.

### 5.6 Urea Oxidation Reaction (UOR)

UOR has emerged as a sustainable and ecofriendly energy solution. Urea (CO(NH_2_)_2_) is abundant, nontoxic, and a high‐energy‐density source with an energy content of 16.9 MJ L^−1^. Typically occurring in alkaline conditions, the UOR engages in a six‐electron‐proton (or hydroxyl) coupled transfer step, as shown below.^[^ ^115^ ^]^

(17) $$\text{CO}{\left({\text{NH}}_{2}\right)}_{2}+6{\text{OH}}^{-}\to N_{2}+{\text{CO}}_{2}+5H_{2}O+6e^{-}\;(E_{0}=0.37\text{ V vs. RHE})$$

Despite its lower thermodynamic equilibrium potential compared to the OER, the UOR suffers from high overpotentials (about 1 V) due to its complex multielectron transfer process.^[^ ^116^ ^]^ This high overpotential necessitates the development of efficient catalysts to lower energy barriers and accelerate reaction kinetics. The UOR mechanism involves several steps, including the adsorption of urea on the catalyst surface and a series of proton‐coupled electron transfer steps, which lead to the breaking of C—N and N—H bonds. However, the detailed mechanism remains unclear, and the six‐electron process is complicated, often resulting in many side products related to NH_*x*_ and NO_*x*_ species. Notably, NO_*x*_ is a common byproduct, which complicates the reaction and reduces efficiency. In the most‐studied Ni‐based catalysts, the formation of NiOOH at high potentials (around 1.36 V) is crucial as it serves as the active site for UOR.^[^ ^117^ ^]^ The pathway on NiOOH surfaces involves key intermediates such as *CON, *CO–NH, and *COO, with the PDS being the desorption of the last *COO intermediate to form CO_3_^2−^ in the electrolyte.^[^ ^115^, ^117^, ^118^ ^]^ This strong binding to Ni^3+^ active sites results in high activation energies, leading to sluggish reaction kinetics and low catalytic activity.^[^ ^119^ ^]^

To address the challenges in UOR and improve selectivity and activity, various catalysts have been explored. Noble metals like Ti, Pt and Ru have shown effectiveness, but their high cost limits widespread application.^[^ ^120^ ^]^ Recent focus has shifted toward non‐noble metal catalysts, such as hydroxides, oxides, sulfides, and phosphides, which offer more cost‐effective solutions but still face issues like high overpotentials and inadequate stability.[^117^, ^121^] Heterostructured catalysts, composed of multiple phases or components, are particularly important for increasing selectivity and activity due to their unique electronic and structural properties. These advancements highlight the potential of UOR as a sustainable alternative in energy conversion and storage technologies, particularly with ongoing efforts to develop more efficient and durable catalysts.^[^ ^122^ ^]^ Specifically, researchers have been using a combination of different 2D materials such as metal dichalcogenides to enhance the efficiency of UOR due to high‐flux electron transfer at the interface.^[^ ^123^ ^]^ Similarly, researchers have emphasized manipulating surface charge redistribution for UOR activity, adapting Schottky junctions for electrocatalysis from semiconductor physics, and promoting further electron transfer for UOR. These findings underscore the significant role of material modification, particularly through sulfur integration, in advancing catalytic efficiency and robustness required for UOR processes.

**Figure 9:**
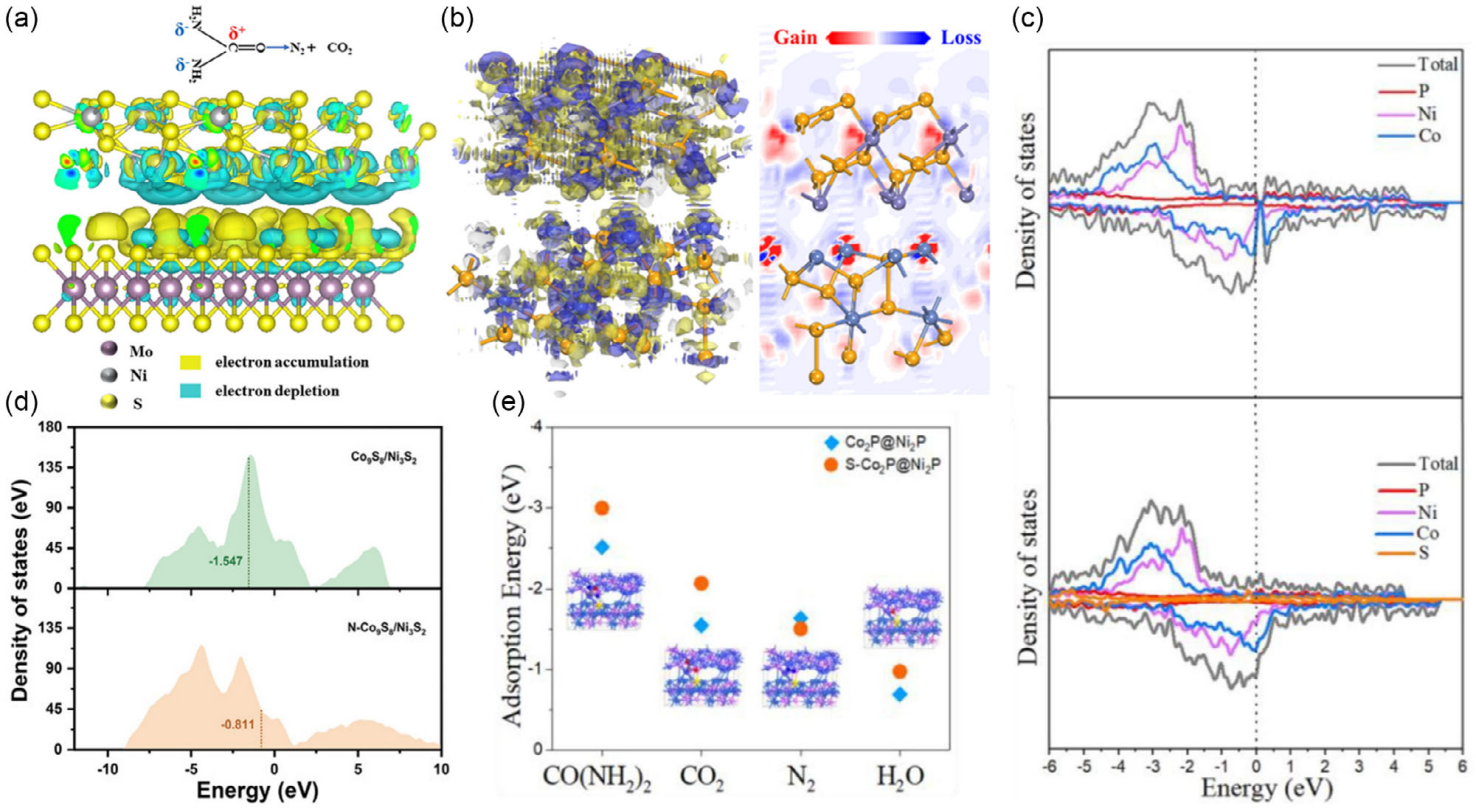
Electronic structure and activity analysis on UOR heterostructured catalyst. a) CDD in the heterostructure of MoS_2_ and Ni_3_S_2_. Reproduced with permission.^[^ ^125^ ^]^ Copyright 2022, Elsevier. b) CDD stereograms of NiSe_2_/FeSe_2_. (left) Yellow and blue contours represent the charge depletion and accumulation, respectively (right) The corresponding sectional drawing of (left). Reproduced with permission.^[^ ^127^ ^]^ Copyright 2021, Elsevier. c) DOS of Co_2_P@Ni_2_P and S‐Co_2_P@Ni_2_P. Reproduced with permission.^[^ ^128^ ^]^ Copyright 2022, Elsevier. d) DOS of Co_9_S_8_/Ni_3_S_2_ and N‐Co_9_S_8_/Ni_3_S_2_ with *d*‐band center. Reproduced with permission.^[^ ^129^ ^]^ Copyright 2023, WILEY‐VCH GmbH. e) Comparison of calculated adsorption free energies of CO(NH_2_)_2_, CO_2_, N_2_, and H_2_O on Co_2_P@Ni2P and S‐Co_2_P@Ni_2_P. Reproduced with permission.^[^ ^128^ ^]^ Copyright 2022, Elsevier.

Exploring the intricate dynamics of charge redistribution at the interface sheds light on the catalytic mechanisms within heterostructured catalysts for UOR. Li et al. emphasized that urea had a unique molecular structure, featuring both an electron‐donating group (amino) and an electron‐withdrawing group (carbonyl), which needs a solid‐state catalyst with a Janus charge distribution surface to facilitate the activation pathway. However, how to precisely manipulate the distribution of positive and negative charges for this specific catalytic process is still not cultivated. With this in mind, they designed a CoS_2_/MoS_2_ heterostructured catalyst, where CoS_2_ is metallic and MoS_2_ is semiconductive. Due to electrostatic interactions, the electron‐donating carbonyl tended to be intensively adsorbed on MoS_2_ side, whereas the electron‐donating group of amino was prone to be adsorbed on CoS_2_ sites. As a result, the interaction between CoS_2_ and MoS_2_ facilitated the fracture of the C—N bond in the urea molecule and promoted its decomposition into CO_2_ and N_2_.^[^ ^124^ ^]^ Similarly, Ren et al. proposed that urea, composed of carbonyl and amino groups, tended to be adsorbed on the electron‐rich side. Thus, CDD not only illustrated electron transport but also identified regions where urea was likely to adsorb. They calculated CDD and mapped it to highlight the electron transfer from Ni_3_S_2_ to MoS_2_, especially at the interface (**Figure** **9**a).^[^ ^125^ ^]^ Liu et al. also suggested that UOR is likely to occur at the interface. They calculated the CDD at the Ni_3_S_2_/Ni_3_P interface, exhibiting that electrostatic interactions allowed electron‐withdrawing carbonyl group of urea to adsorb onto the Ni atoms on the Ni_3_S_2_ side, while the electron‐donating amino group was prone to be adsorbed on the Ni atoms at Ni_3_P side.^[^ ^126^ ^]^ Ni et al. calculated CDD and performed the Bader charge analysis on NiSe_2_/FeSe_2_ to indicate charge exchange at the interface, thus resulting in electron accumulation on the NiSe_2_ side and electron depletion on the FeSe_2_ side (Figure 9b).^[^ ^127^ ^]^ As expected, when water and urea oxidation took place on the surface of *p*–*p* semiconductor heterojunction, the positively charged FeSe_2_ surface favored the adsorption of electron‐rich OH^−^ and the amino group of urea due to the electrostatic interaction, while the electron‐deficient carbonyl group of urea was inclined to be adsorbed on the negatively charged NiSe_2_ surface. Collectively, the utilization of charge density redistribution to ensure constructed heterojunction can facilitate charge exchange at the interface, delineating electron‐rich and electron‐poor regions. This strategic analysis is pivotal in identifying electron‐rich regions as the primary sites for urea adsorption, highlighting the intricate interplay of electronic properties in enhancing catalytic efficiency.

Moreover, PDOS can be utilized to investigate and reveal key insights into the catalytic processes of heterostructured catalysts used in UOR. Peng et al. employed PDOS analysis on NiCo_2_S_4_ and NiMn‐LDH. The PDOS resulting for NiCo_2_S_4_@NiMn‐LDH around the Fermi level was continuous and significantly higher than that of pure NiMn‐LDH, confirming enhanced electron transfer capability due to its more metallic properties. The analysis revealed that the *d*‐orbital of Co and *p*‐orbital of S were responsible for the increased PDOS around the Fermi level, indicating high electrical conductivity. The heterostructure's construction promoted hybridization between the *d*‐orbital of Ni in NiMn‐LDH with the adjacent *d*‐orbital of Co and *p*‐orbital of S in NiCo_2_S_4_, underscoring the synergistic relationship between NiCo_2_S_4_ and NiMn‐LDH.^[^ ^123^ ^]^ Additionally, Yuan et al. enhanced HER and UOR activity by introducing sulfur in a Co_2_P@Ni_2_P core–shell heterostructure. Sulfur doping at the interface caused electrons to move from Co_2_P to Ni_2_P, boosting the reaction efficiency. PDOS analysis showed the creation of new electronic states by hybridization of S 2*p* with Co 3*d*, Ni 3*d*, and P 2*p* orbitals around the Fermi energy level, increasing electron conductivity (Figure 9c).^[^ ^128^ ^]^ Furthermore, Xie et al. examined the PDOS of *N*‐doped Co_9_S_8_/Ni_3_S_2_, showing that the *d*‐band center shifted from −1.547 to −0.811 eV after *N* doping, which improved UOR activity (Figure 9d).^[^ ^129^ ^]^ Overall, PDOS analyses illustrate that heterostructured electrocatalysts can induce new electronic states and enhance electron conductivity, thus playing a pivotal role in improving UOR activity.

Urea electrocatalysis is a complex process with an intricate and not fully understood mechanistic pathway. In fact, depending on different catalysts, different side reactions can be formed, resulting in different side products. Thus, the adsorption energy of urea is frequently used as a key descriptor to better characterize UOR activity. Urea's carbonyl group, being electrophilic, is attracted to electron‐rich areas, while the amino groups, being nucleophilic, interact with electron‐poor sites. Peng et al. reported enhanced urea adsorption energy can improve UOR activity of NiCo_2_S_4_ and NiMn LDH. DFT results showed that the adsorption energy of urea on the surface of NiCo_2_S_4_@NiMn LDH was −2.91 eV, while that of NiCo_2_S_4_ was −1.76 eV and NiMn LDH was −0.94 eV. They asserted that the adsorption capability of urea molecules on the surface and the subsequent dissociation of the intermediates have a significant impact on the UOR activity. The enhanced adsorption energy of urea indicates that engineering the interface of the heterostructure improves both the adsorption and activation of urea molecules, addressing the sluggish dynamics of the UOR.^[^ ^123^ ^]^ Similarly, Yuan et al. calculated adsorption energies of intermediates such as CO(NH_2_)_2_, H_2_O, N_2_, and CO_2_ on *S*‐doped Co_2_P@Ni_2_P. For urea, carbonyl and amino‐group of urea molecules tend to be adsorbed on the electron‐rich side and electron‐deficient side, respectively. CO(NH_2_)_2_, H_2_O prefer adsorption on S‐Co_2_P@Ni_2_P (−3.00, −0.97 eV) than Co_2_P@Ni_2_P (−2.52, −0.68 eV), where the produced N_2_ gas can be desorbed more easily (−1.50, −1.64 eV) (Figure 9e). Such results highlight that *S*‐doping at the interface of S‐Co_2_P@Ni_2_P induces the electron redistribution, which thermodynamically favors the adsorption of reactants and desorption of products for facilitated UOR kinetics.^[^ ^128^ ^]^ These findings emphasize urea adsorption can be controlled with utilizing heterostructured catalysts, which is crucial for enhancing UOR activity.

## 6 Machine Learning Approaches for Advanced Heterostructure Analysis

Machine learning (ML) has demonstrated its advantages in enhancing computational efficiency, alleviating computational costs, and accelerating material discovery.^[^ ^130^ ^]^ In the field of materials science and catalysis, ML has been extensively utilized for broad candidate exploration, identifying key descriptors based on feature importance, proposing intrinsic descriptors, and developing ML potential with significantly reduced computational time.^[^ ^131^ ^]^ To effectively develop and discover novel materials using ML, in‐domain knowledge that considers chemical and physical properties is essential. In addition, integrating DFT‐derived data improves both accuracy and efficiency of ML models.^[^ ^132^ ^]^ Therefore, understanding critical insights from DFT‐based results such as structural modeling, catalytic stability/activity, and electronic structure, as previously introduced, can further enhance the quality of ML frameworks. The growing importance of ML has extended into the computational studies of heterostructures.

Heterostructure systems require complex approaches with the inherent interactions of two or more materials, which can affect the structural and electronic characteristics of heterostructures and catalytic activity.^[^ ^133^ ^]^ ML‐assisted heterostructure studies have particularly focused on 2D material‐based systems, with significant emphasis on vdW heterostructures, where each layer retains its original structure.[^9^, ^19^, ^134^] These heterostructures provide immense potential for novel material development due to their tunable bandgaps, electronic structures, and diverse active sites, which arise from the interactions between materials and the strain effects within the structure.^[^ ^133^ ^]^ Furthermore, vdW heterostructures can maximize their catalytic performance by forming superlattices or Moiré lattices at specific twist angles between layers.^[^ ^135^ ^]^ Such vast optimization and exploration space makes ML a powerful tool for accelerating material discovery and property prediction in 2D material‐based heterostructures.

ML has been effectively employed to enhance the efficiency of modeling heterostructures, addressing their inherent complexities and enabling more accurate and efficient simulations. Celis et al. investigated 2D vdW heterostructures composed of *γ*‐PC (phosphorus carbide) and WS_2_, utilizing the complete bilayer basis deviation (CBBD) algorithm. This ML approach overcame the challenges of reaching local minima and forming metastable structures in periodic boundary conditions during DFT modeling. They aligned at least one atomic site in each layer with identical *x* and *y* coordinates, ensuring the construction of rationally finite supercells and minimizing structural irregularities. Additionally, a consistent initial interlayer distance of 3 Å was applied, with 20 Å vacuum to eliminate interference between supercells. Using the CBBD algorithm, they generated 18 123 vdW heterostructures, encompassing isotropic strain, anisotropic strain, and intralayer shear strain models. Notably, this study demonstrated that the input unit cell did not need to adhere to a uniform hexagonal structure, mitigating the difficulties in conventional DFT modeling. They selected 45 isotropically strained *γ*‐PC/WS_2_ vdW vertical heterostructures from an extensive ML‐generated dataset and analyzed them using DFT calculations. In the six systems containing the fewest number of atoms, the interlayer spacing ranged from 3.27 to 3.77 Å, showing a linear relationship between reduced spacing and increased binding energy. However, in larger systems, the spacing stabilized in a narrow range from 3.52 to 3.56 Å, with consistent binding energies. Also, out‐of‐plane corrugation became more pronounced at lower twist angles (0°–30°), though no distinct correlation with binding energy was observed. By accommodating a diverse range of strain configurations, the CBBD algorithm effectively expanded the pool of realistic vdW heterostructure candidates and allowed for more comprehensive simulation of the system.^[^ ^136^ ^]^

In addition, ML has simulated the dynamic surface of heterostructures under electrochemical conditions. Wan et al. constructed a NiO_*x*_H_*y*_@Pt heterostructure in an alkaline electrolyte, using a grand canonical genetic algorithm (GCGA) algorithm for surface modeling.^[^ ^137^ ^]^ The genetic algorithm is useful for global optimization, particularly in molecular and structural modeling, as it facilitates the exploration of complex potential energy surfaces.^[^ ^138^ ^]^ Through a bond length distribution algorithm, the researchers generated random initial structures, which were subsequently optimized using the GCGA algorithm to simulate the intricate electrochemical environment of NiO_*x*_H_*y*_.^[^ ^139^ ^]^ The optimized structure featured a Pt core encapsulated by a highly ordered Ni(OH)_2_ shell, which enhanced water decomposition, electrolyte‐core separation, and overall stability. The NiO_*x*_H_*y*_ structure, identified as the global minimum, exhibited various adsorbed states such as OH, water, and bridging oxygen. Using the algorithm‐optimized structure, they conducted DFT calculations to gain further insights into the electronic properties and catalytic behavior of the NiO_*x*_H_*y*_@Pt heterostructure. The calculations revealed charge transfer from Pt to NiO_*x*_H_*y*_, which was corroborated by X‐ray absorption near‐edge structure analysis. The reduced electron density on Pt facilitated H adsorption. As a result, the NiO_*x*_H_*y*_@Pt heterostructure created a favorable local chemical environment for the HER.^[^ ^137^ ^]^ These approaches, combining algorithm‐driven structural modeling and DFT calculations, underline the synergistic effects in uncovering complex phenomena associated with heterostructure formation and investigating dynamic surface behaviors under realistic electrochemical conditions.

**Figure 10:**
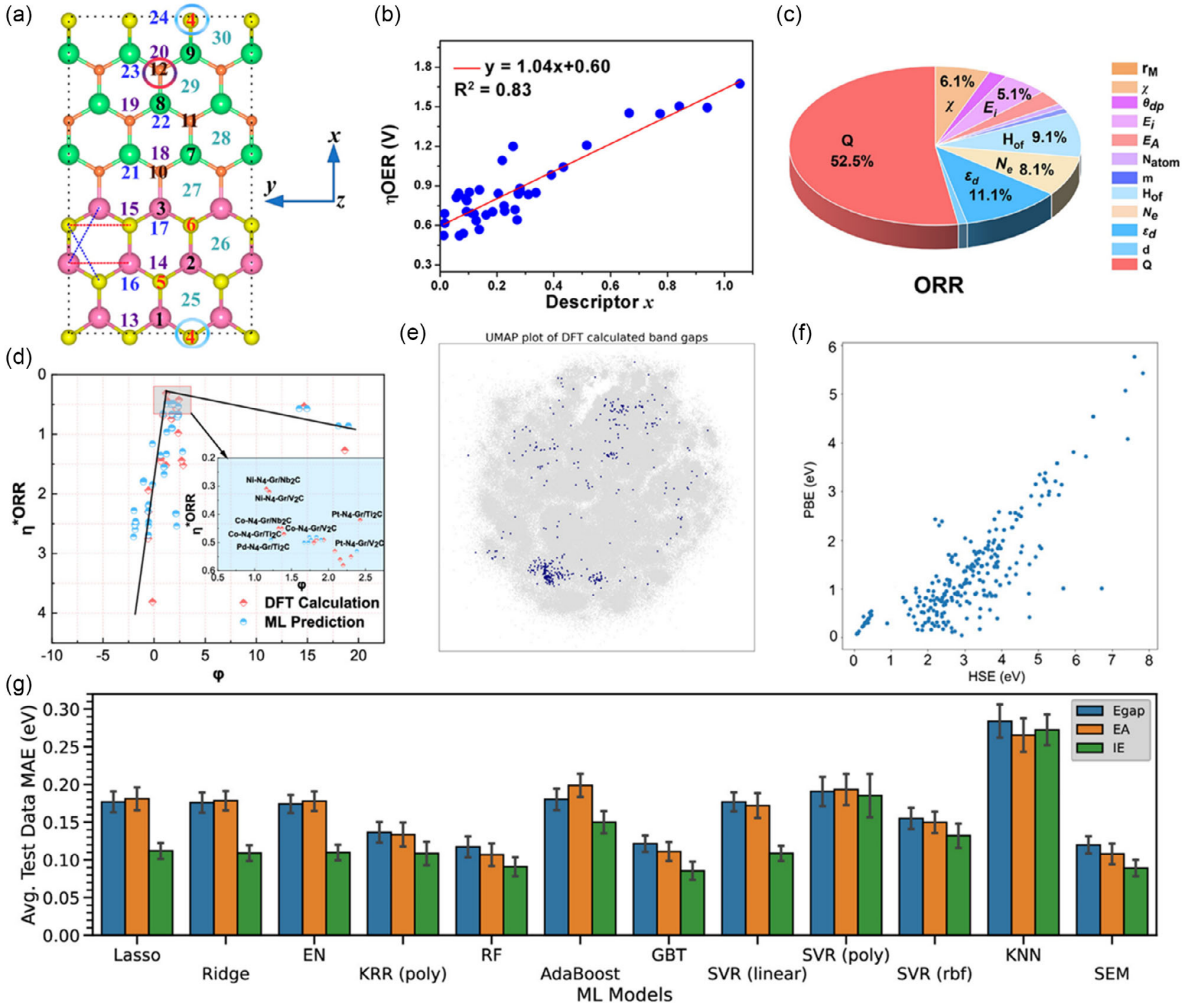
a) Visualization of 30 hydrogen adsorption sites on a 2D lateral heterostructure. Reproduced with permission.^[^ ^140^ ^]^ Copyright 2023, American Chemical Society. b) Prediction of OER overpotentials based on the LASSO performance descriptor (*x*). Reproduced with permission.^[^ ^58^ ^]^ Copyright 2020, American Chemical Society. c) Feature importance analysis of the 12 features for ORR in the RFR model. d) ORR overpotential prediction based on the intrinsic catalytic activity descriptor (*φ*). Reproduced with permission.^[^ ^145^ ^]^ Copyright 2023, American Chemical Society. e) UMAP showing DFT bandgap calculations (dark blue) across a 2.2 million dataset (light gray). f) Correlation between bilayer bandgaps calculated by GGA‐PBE and HSE06. Reproduced under the terms of the Creative Commons CC BY license.^[^ ^148^ ^]^ g) Average *k*‐fold crossvalidation MAE of the test data across diverse ML models. The black line indicates the standard deviation of the MAE. Reproduced with permission.^[^ ^150^ ^]^ Copyright 2022, American Chemical Society.

By integrating DFT insights with ML, efficient ML models can evaluate extensive heterostructure materials, even from limited datasets. These models, trained on DFT‐derived data, can identify key features, establish meaningful correlations, and predict catalytic performance across diverse material systems. ML model accuracy can be assessed using metrics such as coefficient of determination (*R* ^2^), mean absolute error (MAE), mean‐squared error (MSE), and root mean squared error (RMSE). Furthermore, feature importance analysis, Shapley additive exPlanations (SHAP), and Pearson and Spearman correlation coefficients provide deeper interpretations of key contributing factors. For example, Pham et al. developed an ML model to predict HER activity across 30 adsorption sites in lateral heterostructures using DFT‐derived datasets. Group A consisted of TMDs such as MoS_2_/WS_2_ and MoTe_2_/WSe_2_, while Group B combined dichalcogenides with MX compounds containing O or N, including MoS_2_/ZnO and NbS_2_/GaN. Bandgap calculations indicated that Group A displayed semiconducting properties, while Group B exhibited metallic characteristics, primarily influenced by the PDOS of Mo and Nb near the Fermi level. These results suggested the potential of MoS_2_, MoSe_2_, NbS_2_, and NbSe_2_ combined with ZnO, GaN, and AlN as promising HER catalysts due to their enhanced electrical conductivity. Hydrogen adsorption energy calculations were conducted across 30 adsorption sites. Group A interfaces displayed positive Gibbs free energies, indicating unfavorable hydrogen adsorptions, whereas Group B showed negative Gibbs free energies, reflecting higher HER catalytic potential. Among these sites, the most favorable active sites were identified as S/Se/N/O top sites and interfacial positions 4 and 12 (**Figure** **10**a). Specifically, the S and Se top sites at position 4 demonstrated superior HER activity in NbS_2_/GaN and NbSe_2_/ZnO heterostructures, while the O and N top sites at position 12 showed enhanced activity in MoS_2_/ZnO and MoSe_2_/ZnO systems. To construct an efficient ML model, 46 features were derived and optimized using a gradient boosting‐based regression and classification approach with light gradient boosting machine (LightGBM). The trained model predicted HER activity across 30 adsorption sites, successfully discovering TaS_2_/ZnO and TaS_2_/AlN as promising HER catalysts from an additional set of Group B heterostructures. A broader trend analysis revealed that active sites within the ±0.25 eV Gibbs free energy range were predominantly associated with top sites, emphasizing their catalytic preference. Validation through 12 additional DFT calculations confirmed the strong predictive accuracy of the ML model. SHAP analysis identified average atomic ionization potential, periodic column, and positional attribute as the most significant features, with SHAP values of +0.17, +0.13, and +0.12, respectively. However, Pearson and Spearman correlations revealed inconsistencies in their ranking, highlighting the need for multifaceted analysis when interpreting feature contributions in ML models.^[^ ^140^ ^]^

Similarly, Jyothirmai et al. explored the HER activity of 180 g‐C_3_N_4_/MX_2_ heterostructures (M = Mo, W; X = S, Se, Te) by intercalating ten TMs across three distinct sites and performing hydrogen adsorption energy calculations. Adsorptions on S atoms in the MX_2_ layer resulted in positive Gibbs free energies, indicating unfavorable adsorptions. In contrast, C and N sites on the *g*‐C_3_N_4_ layer exhibited negative Gibbs free energies, suggesting a higher preference for hydrogen adsorption. Heterostructures with optimal adsorption energies ranging from −0.3 to +0.3 eV over 50% involved Sc and Ti intercalation. Out of 540 possible adsorption configurations, 270 random structures were selected for ML training. Ten ML algorithms, including random forest regression (RFR), gradient boosting gradient (GBR), support vector regression (SVR), linear regression, and kernel ridge regression (KRR), were evaluated. RFR, optimized through hyperparameter tuning, achieved the highest predictive performance (*R* ^2^: 0.957, MAE: 0.118 eV). SHAP analysis indicated hydrogen adsorption on C sites and MX layers as the most influential features, providing general insights into the preferential hydrogen adsorption sites in g‐C_3_N_4_/MX_2_ heterostructures.^[^ ^141^ ^]^ Furthermore, Liu et al. adopted an artificial neural network (ANN) to screen 27 TMs for HER activity in Ag(M) catalysts.^[^ ^142^ ^]^ A dataset of 170 hydrogen adsorption Gibbs free energy values was utilized to train the ML model.^[^ ^143^ ^]^ Using the maximum information coefficient, ten critical features were extracted, with electron affinity (EA) represented as the most important feature, contributing 27.3%. The ANN model achieved high predictive accuracy (*R* ^2^: 0.87, RMSE: 0.03 eV) and discovered Ag(Ni), Ag(Mn), Ag(Co), and Ag(Cu) as promising candidates. Experimental validation focused on Ag(Ni), revealing that charge redistribution played a pivotal role in enhancing HER activity. DFT calculations confirmed that Ni incorporation enhanced HER activity through thermoneutral hydrogen adsorption, an upshifted *d*‐band center, and charge redistribution. This synergistic effect resolved the weak adsorption on Ag sites.^[^ ^142^ ^]^ These findings highlight the potential of integrating ML and DFT methodologies to accelerate HER catalyst discovery and suggest a robust framework for developing next‐generation catalysts.

ML can also construct intrinsic descriptors by integrating structural, chemical, and physical attributes to predict catalytic performance and identify key features. These descriptors streamline catalyst screening, reduce computational costs, and provide generalized frameworks for catalytic performance prediction. The least absolute shrinkage and selection operator (LASSO) is particularly effective for optimizing linear predictive models by focusing on the most influential features while eliminating less relevant ones, enhancing both accuracy and interpretability.^[^ ^144^ ^]^ Ge et al. investigated eight dichalcogenide‐based vdW heterostructures across six rotation angles in 60° increments. The adsorption energy calculations for OH, O, OOH, and H species focused on nonmetal sites on the basal plane, where adsorbates tend to migrate. For OER, MoTe_2_/WTe_2_ at 0° rotation exhibited an overpotential of 0.16 V, while for HER, the MoSe_2_/WSe_2_ at a 240° rotation achieved excellent activity with an overpotential of 0.01 V. By analyzing 192 adsorption energy points across 48 configurations, they confirmed a scaling relationship between OH, O, and OOH adsorption energies, providing a guideline for predicting catalytic trends using volcano plots. However, despite the utility of scaling relationships in identifying optimal catalysts, their reliance on extensive DFT calculations remains computationally demanding. To address this challenge, they aimed to derive intrinsic descriptors capable of physically predicting catalytic activity without exhaustive DFT computations. Instead of relying on commonly used atomic and chemical properties, they selected rotation angle, interlayer spacing, bond length, and bandgap ratio as input parameters for the LASSO model. By systematically analyzing these descriptors, they generated 257 703 relational equations and derived an optimal linear relationship to predict the theoretical overpotentials for OER and HER based on the intrinsic descriptor. The resulting model exhibited R^2^ values of 0.83 for OER and 0.80 for HER, indicating robust predictive accuracy (Figure 10b). As a result, MoTe_2_/WTe_2_ at a 300° rotation emerged as the most promising bifunctional catalyst, with overpotentials of 0.03 V for HER and 0.17 V for OER, respectively.^[^ ^58^ ^]^ This study not only identified an optimal heterostructure but also demonstrated the potential of intrinsic descriptors based on physical parameters to predict catalytic performance efficiently.

Additionally, Chen et al. analyzed 78 heterostructures composed of M–N_4_–graphene (M‐N_4_‐Gr) and MXene substrates, where the TMs (M) spanned from Ti to Au, excluding Tc, Cd, and Hg, and the MXene substrates included Ti_2_C, Nb_2_C, and V_2_C. Among these, 28 structures were evaluated using DFT, and 50 additional structures were assessed via ML models, enabling rapid catalyst screening. DFT calculations revealed Ni–N_4_–Gr/Nb_2_C and Ni–N_4_–Gr/V_2_C as efficient ORR catalysts, while Ru‐N_4_‐Gr/Nb_2_C demonstrated superior OER activity. Additionally, Co–N_4_–Gr/V_2_C exhibited bifunctional catalytic capabilities for both ORR and OER. AIMD simulations confirmed the dynamic stability at 500 K for 5 ps, with no observed bond breaking or formation. Further electronic structure analysis highlighted that promising candidate possessed *d*‐band centers between −1 and −2.2 eV, ensuring a moderate adsorption strength. Bader charge analysis revealed V_2_C substrates as favorable due to their highest charge loss. To identify key catalytic features, 12 atomic structural, electronic structural, and environmental structural features were evaluated across five ML models, with the RFR model demonstrating superior predictive performance. Feature importance analysis showed that charge transfer amount (Q) accounted for 52.5% and the *d*‐band center of active metal ($\varepsilon_{d}$ ) contributed 11.1% for ORR overpotential (Figure 10c). For OER overpotential, the number of *d* and *p* electrons ($\theta_{dp}$) was the most influential feature at 26%, followed by charge transfer amount (*Q*) at 23%, and first ionization energy (IE) ($E_{i}$) at 15.5%. An intrinsic descriptor (*φ*) was established through Pearson correlation analysis, identifying positive correlations with charge transfer amount (*Q*), *d*‐band center of active metal ($\varepsilon_{d}$), and atomic radii (*r*). Conversely, negative correlations were observed with oxide formation enthalpy ($H_{o,xf}$), number of outermost electrons ($N_{e}$), electronegativity (*χ*), and the number of *d* and *p* electrons ($\theta_{dp}$ ). Using this descriptor, the study effectively predicted OER and ORR overpotentials for new structures without requiring DFT calculations, reducing computational time from 48 GPU h (the number of image processing unit hours) to 32 s per calculation (Figure 10d).^[^ ^145^ ^]^ This approach underscored the scalability of integrating DFT and ML methodologies and demonstrated the efficiency of intrinsic descriptors for guiding catalyst design and optimization.

Similarly, Bai et el. explored 153 MN_4_–graphene (MN_4_‐Gra)/MXene (M_2_NO) heterostructures to design efficient bifunctional electrocatalysts for OER and ORR. DFT calculations were conducted on 19 selected MN_4_‐Gra/M_2_NO structures and revealed CoN_4_‐Gra/Ti_2_NO as an efficient bifunctional catalyst with ORR and OER overpotentials of 0.37 and 0.30 V, respectively. AIMD simulations at 300 K for 3 ps confirmed the structural integrity under operating conditions. For ML model training, unsuitable heterostructures such as TiN_4_–Gra/M_2_NO and MN_4_–Gra/Cr_2_NO were excluded. Among several ML models, the GBR model outperformed other RFR, SVR, and symbolic regressions (SR) with the highest accuracy. SR was specifically applied to establish the intrinsic descriptor for predicting ORR and OER overpotentials, incorporating covalent radius, IE, and electronegativity. The analysis revealed that ORR overpotentials were predominantly influenced by TMs of the MXene layer (M_2_) and OER overpotentials were primarily governed by TMs in the MN_4_–Gra layer (M_1_). Model validation using ten random heterostructures confirmed the GBR model's predictive accuracy, achieving the lowest MAE among DFT, SR, and GBR methods. Additionally, the ML approach suggested RuN_4_‐Gr/W_2_NO as a bifunctional catalyst with ORR overpotential of 0.39 V and OER overpotential of 0.45 V.^[^ ^146^ ^]^ This study showed that while SR identifies key intrinsic features, the GBR model predicted more accurate catalytic activity.

2D vdW heterostructures offer tunable bandgaps, enabling precise control over electronic and optical properties.^[^ ^133^ ^]^ Despite advancements in exchange‐correlation functionals like generalized gradient approximation (GGA) and hgeyd‐scuseria‐ernzerhof (HSE06), bandgap calculations remain computationally intensive and often suffer from underestimation.^[^ ^147^ ^]^ To address these limitations, ML approaches have been performed to gain insights into bandgap behavior through theoretical modeling. Fronzi et al. developed a large‐scale database comprising ≈2.2 million bandgap datasets for vdW heterostructures using ML.^[^ ^148^ ^]^ They utilized the 2DMatPedia database and selected 109 random bilayer structures with intralayer energies below −1.0 eV Å^−2^, lattice mismatches below 2%, and fewer than 200 atoms per unit cell.^[^ ^149^ ^]^ Bandgap values were computed using the HSE06 hybrid functional known for its accuracy but requiring 5–7 times more computational resources than GGA with the perdew‐burke‐ernzerhof (PBE) calculations. To enhance computational efficiency, an active learning framework employing a Bayesian neural network (BNN) was adopted. The training began with a small labeled dataset, and was iteratively refined through *k*‐means clustering algorithm, selecting high‐uncertainty structures, validating them with HSE06 calculations, and incorporating them into the training dataset. Uniform manifold approximation and projection (UMAP) analysis ensured a uniform distribution of data points, illustrating that structures with similar physicochemical properties are naturally clustered closely (Figure 10e). The dataset was categorized by optical spectrum ranges, and a correlation between HSE06 and GGA–PBE bandgaps showed Pearson and Spearman coefficients of 0.68 and 0.60, respectively (Figure 10f). However, the feature importance analysis indicated that the GGA–PBE bandgap had a relatively low influence (0.13) on the BNN model and LASSO regression exhibited that other structural descriptors played a more significant role. Interestingly, when the BNN model was trained exclusively with 425 randomly selected structures without incorporating HSE06‐derived data, the *R* ^2^ value was 0.51 with insufficient predictive accuracy. This emphasized the importance of incorporating high‐accuracy HSE06 data in the initial training dataset to improve predictive performance while balancing computational efficiency.^[^ ^148^ ^]^ Therefore, this study also demonstrated that, despite advancements in ML methodologies, DFT calculations remain indispensable for generating high‐quality training datasets.

Furthermore, Willhelm et al. employed a stacked ensemble method (SEM) to integrate three distinct ML models, effectively combining their strengths to predict the properties of heterostructures based on material configurations.^[^ ^150^ ^]^ Utilizing the Computational 2D Materials Database, which includes high‐throughput DFT and green's function G and the screened coulomb interaction W (GW) many‐body perturbation theory calculations, they applied five selection criteria and excluded magnetic materials.^[^ ^151^ ^]^ They identified 181 unique monolayers derived from seven 2D prototypes, MoS_2_, CdI_2_, GaS, GaSe, GeSe, hBN, and graphene. From these monolayers, 16 290 possible heterostructures were produced, and those with lattice mismatches below 3% were selected, resulting in 1950 AA‐stacked and 1950 AB‐stacked vertical heterostructures. High‐throughput DFT calculations revealed that AB‐stacked structures exhibited shorter interlayer distances and more stable binding energies. Structures with interlayer distances below 2.5 Å were excluded to avoid potential chemical bonding and ensure the retention of vdW interactions. Additionally, bilayers exhibiting metallicity or Type III band alignments were filtered out, leaving 595 bilayers for ML model training. A LASSO estimator was employed to reduce the initial 91 features to 59 for bandgap prediction and to 55 for both EA and IE prediction. LASSO coefficient analysis revealed that the CBM and VBM contributions from individual monolayers were critical in determining the bilayer bandgaps. To build robust predictive models by balancing the strengths and weaknesses of various ML models, SEM integrated multiple gradient boosted trees (GBT), ridge regression, linear SVR, and polynomial KRR. The SEM achieved MAEs of 0.122 eV for bandgap (*E*_g_), 0.112 eV for EA, and 0.09 eV for IE (Figure 10g). This approach outperformed conventional bandgap predictions based on Anderson's rule, which assumes no interlayer interactions or charge transfer. Beyond bandgap prediction, ML models also predicted interlayer distance and binding energy. Using LASSO estimators, the features were reduced to 64 for interlayer distance and to 50 for binding energy. Among the evaluated ML models, the SEM approach demonstrated the lowest MAE, highlighting its superior predictive performance.^[^ ^150^ ^]^ This study underscores the potential of SEM ML frameworks to deliver accurate and reliable predictions for 2D vdW heterostructure properties.

## 7 Conclusion and Perspectives

Computational approaches to modeling heterostructured electrocatalysts offer significant potential for advancing sustainable energy technologies. This review highlights key challenges and proposes systematic approaches, including accurate selection of modeling types, managing lattice strain, determining stability, simulating surface conditions, and identifying active factors through electronic structure properties.

Key perspectives for future research include the following. 1) Selecting an appropriate modeling type is essential for accurate simulations of heterostructured electrocatalysts. This review classifies computational modeling into three types: vertical, semivertical, and lateral heterostructure. Vertical heterostructure modeling is ideal for studying full interlayer interactions, semivertical heterostructure modeling is effective for examining edge sites and partial interface coverage, and lateral heterostructure modeling facilitates detailed analysis of horizontal interactions and active site exposure. These approaches enable precise evaluations of critical factors in heterostructure, contributing to the development of efficient electrocatalysts. 2) Managing lattice strain and predicting synthesis feasibility in computational modeling are important, as lattice strain directly affects catalytic activity and stability. Extensive reference data from similar material groups is essential for this process. By actively sharing lattice strain data, researchers can establish strain standards for different materials, enabling precise application of tensile and compressive strain effects. This also helps to minimize unwanted distortions in heterostructures. Such efforts will accelerate high‐quality computational research. In addition, heterostructure modeling relies heavily on HRTEM, making it important to develop methods that predict structural parameters like interfacial angles, distances, and facets for advancing computation‐driven research. 3) To improve accuracy, it is essential to evaluate the stability and simulate the surface conditions of heterostructures. Calculating (normalized) interfacial binding energy helps determine the thermodynamic favorability of heterostructure formation, while (vacancy) formation energy reflects the surface states that emerge during electrochemical reactions. Incorporating conditions like oxidation, phase transitions, and vacancy formation into these calculations can enhance the predictive accuracy of computational models, aligning them more closely with the behavior of real electrocatalysts. 4) In electrochemical catalysis, activity is influenced by multiple steps, making it essential to analyze reaction pathways at various active sites. Scaling relations enable computational models to provide efficient insights by linking these pathways. For reactions with unclear mechanisms, such as NRR and UOR, adsorption energy serves as a crucial descriptor. Key factors impacting catalytic performance include CDD, ELF, DOS, and work function, all of which require comprehensive analysis to understand their combined effects. Moreover, in heterostructures, both the intrinsic properties of the materials and the newly formed interfaces must be carefully examined to optimize overall catalytic performance. This approach provides valuable insights into understanding the role of each material within the heterostructure, helping to clarify their contributions to the overall system. 5) Advancements in ML have brought significant breakthroughs to materials science and heterostructure research, offering powerful tools to address the inherent complexity of these systems. This review highlighted various ML approaches applied to 2D materials‐based and vdW heterostructures, demonstrating their potential to optimize structural modeling and accurately predict catalytic performance. By improving computational efficiency and enabling large‐scale modeling, ML is expected to overcome the limitations of traditional methodologies, providing generalized theoretical insights and accelerating the discovery of high‐performance heterostructured electrocatalysts. Moreover, ML is anticipated to extend beyond 2D materials‐based heterostructures, facilitating exploration across a broader range of material groups and more diverse heterostructure configurations. However, despite the growth of ML methodologies, DFT results remain indispensable for generating high‐quality datasets and validating predictions. Therefore, future research will require a balanced integration of ML and DFT to achieve a comprehensive understanding of heterostructure behaviors under practical operating conditions.

## Conflict of Interest

The authors declare no conflict of interest.

## Author Contributions

**Miyeon Kim**: conceptualization, writing—original draft, review and editing. **Kyu In Shim**: conceptualization, writing—original draft, review and editing. **Jeong Woo Han**: conceptualization, supervision, writing—review and editing. **Miyeon Kim** and **Kyu In Shim** equally contributed to this work.
